# Chemical Characterization and Evaluation of Antimicrobial, Antioxidant, and Synergistic Activities of *Teucrium polium* L.: An Integrated Experimental and In Silico Approach

**DOI:** 10.3390/pharmaceutics18030397

**Published:** 2026-03-23

**Authors:** Khalid Zibouh, Brahim Ed-Damsyry, Aziz Drioiche, Mohamed Ed-Dahmouny, Noorah A. Alkubaisi, Mourad A. M. Aboul-Soud, Firdaous Remok, Chaimae Ibbur, Mohamed Radi, Atika Ailli, Sevser Sahpaz, Touriya Zair

**Affiliations:** 1 Research Team of Chemistry of Bioactive Molecules and the Environment, Laboratory of Innovative Materials and Biotechnology of Natural Resources, Faculty of Sciences, Moulay Ismaïl University, B.P. 11201 Zitoune, Meknes 50070, Morocco; k.zibouh@edu.umi.ac.ma (K.Z.); b.eddamsyry@edu.umi.ac.ma (B.E.-D.); mo.eddahmouny@edu.umi.ac.ma (M.E.-D.); f.remok@edu.umi.ac.ma (F.R.); c.ibbur@edu.umi.ac.ma (C.I.); m.radi@edu.umi.ac.ma (M.R.); a.ailli@umi.ac.ma (A.A.); 2 Higher Institute of Nursing Professions and Health Techniques of Fez, Regional Health Directorate Fez-Meknes, EL Ghassani Hospital, Fez 30000, Morocco; 3 Department of Botany and Microbiology, College of Science, King Saud University, Riyadh 11451, Saudi Arabia; nalkubaisi@ksu.edu.sa; 4 Center of Excellence in Biotechnology Research, College of Applied Medical Sciences, King Saud University, Riyadh 11433, Saudi Arabia; maboulsoud@ksu.edu.sa; 5 Specialized Metabolites of Plant Origin, UMRT 1158 BioEcoAgro, JUNIA, University of Lille, F-59000 Lille, France; sevser.sahpaz@univ-lille.fr; 6 University of Liège, 4000 Liège, Belgium; 7 Université de Picardie Jules Verne, F-80025 Amiens, France

**Keywords:** *Teucrium polium* L., limonene, β-eudesmol, epicatechin, synergy, FICI, multidrug-resistant bacteria

## Abstract

**Background/Objectives: ***Teucrium polium* L. is widely used in traditional medicine and has been proposed as a source of antimicrobial adjuvants in the context of antimicrobial resistance. Here, we characterized the essential oil (EO) and polar extracts of *T. polium* and evaluated their antioxidant activity, antimicrobial potency against clinical multidrug-resistant (MDR) isolates, and the interaction of the EO with conventional antibiotics using a chequerboard assay (FICI); further, we investigated in silico molecular interactions with some targets related to resistance. **Methods/Results:** The EO, which was hydrodistilled and subsequently analyzed by GC–MS, is characterized by dominant limonene content (24.13%) and contents of oxygenated sesquiterpenes such as β-eudesmol (10.48%) and α-muurolol (8.10%). HPLC/UV–ESI–MS characterization of the extracts (decoction and Soxhlet) demonstrated that they were rich in polyphenolic compounds and flavonoids, which matched the standard phytochemical characteristics of this species. The extracts exhibited significant reducing capabilities, and the hydroethanolic extract exhibited the highest antioxidant activity (DPPH IC_50_ = 15.41 μg/mL; FRAP EC_50_ = 30.65 μg /mL), while the EO revealed at most moderate capacity in these tests. In antimicrobial assays, the EO inhibited fungi more effectively than the extracts (MIC of 1.17 mg/mL against *Aspergillus niger*; 4.69 mg/mL against *Candida* spp.), while antibacterial MICs for both the EO and extracts were generally high (up to 50 mg/mL). Combination testing nevertheless identified synergistic or additive effects of the EO with selected antibiotics, notably with ceftazidime against ESBL-producing *Escherichia coli* (FICI = 0.141) and *Staphylococcus aureus* (FICI = 0.039) and with amikacin against *Klebsiella pneumoniae* (FICI = 0.313); the EO–ceftriaxone pairing against ESBL *E. coli* was additive (FICI = 0.516). Docking simulations further supported these observations by showing the favorable predicted binding of oxygenated sesquiterpenes, most notably β-eudesmol and α-muurolol (up to −8.6 kcal/mol), to resistance-related targets such as RND efflux pumps, β-lactamases, and porins. **Conclusions:** Taken together, the *in vitro* and *in silico* data suggest that *T. polium* could be explored as a natural antimicrobial option and as an adjuvant to enhance antibiotic activity against multidrug-resistant pathogens.

## 1. Introduction

Microbial resistance to antibiotics is a worldwide health problem with an increasing trend, basically caused by the inappropriate use of these agents and dissemination in bacterial populations of resistance determinants. This troubling situation is compounded by the stagnation in the discovery of new classes of antibiotics since the 1950s [1]. Additionally, the related side effects to chemical antimicrobial agents and the capacity of pathogenic microorganisms to develop resistance against antibiotics have increased the interest of the research community in aromatic and medicinal plants (AMPs), known as alternative sources to natural bioactive molecules with tremendous medicinal potential [2,3].

In this context, some families of aromatic and medicinal plants (AMPs) are considered a valuable source of secondary metabolites, such as terpenoids, polyphenols and flavonoids, that were found to be related with antimicrobial, antioxidant and anti-inflammation activities [4]. In particular, EOs are characterized by a complex composition and multi-target effects that may act at the same time on membrane integrity and permeability, oxidative stress, and some enzymatic activities. This plurality of modes of action is often cited as a possible means to restrict resistance development and positive interaction with classical antibiotics [5].

The *Lamiaceae* family of plants has many species that are high in phenolic compounds, which are renowned for having important medicinal properties [6]. The *Teucrium* genus, which has more than 340 species, is common in sunny areas within this family [7].

*Teucrium polium* L., in Morocco commonly called “Jaada”, is an aromatic perennial species largely used in ethno-medicine throughout Southwest Asia and North Africa, as well as Mediterranean Europe [7]. Its aerial parts are commonly used in folk medicine as digestive, hepatic, metabolic and anti-inflammatory medicament [8]. Recent pharmacological studies evidence multiple biological activities of these traditional uses, such as anti-inflammatory [9], antibacterial [10], antifungal [11], antidiabetic [12], and antioxidant effects [13], and even anticancer prospective use [9].

Phytochemical investigations on this plant showed that *T. polium* is chemically rich in different types of secondary metabolites, such as diterpenes, monoterpenes, sesquiterpenes, polyphenols and strongly antioxidant flavonoids [14]. Over 130 molecules have been described, mostly represented by terpenoids (60%) [15].

The antimicrobial effects of *T. polium* have been reported in many studies. EO of germacrene D and β-pinene showed significant antibacterial effects on *Proteus mirabilis*, *Staphylococcus aureus*, and *Citrobacter freundii*, and antifungal activity against *Trichophyton rubrum* and *Microsporum canis* [16]. Similarly, the essential oil from *T. polium* is originated of Morocco and has been proved to exhibit significant antimicrobial activity against nosocomial infections [10]. In the same context, ethanolic extracts of *T. polium* have also been reported to promote the efficacy of antibiotics, indicating an intriguing modulating potential [17]. Yet the data are heterogeneous given different extraction processes, geographical origins of plant material, and panels of microorganisms challenged, and mechanistic proof is frequently lacking. In fact, most works are confined to essentially in vitro assays and do not relate findings with potential molecular mechanisms of interaction between the main active principles of the extract and bacterial targets associated with resistance/virulence. In this framework, in silico methods, including molecular docking, can be used as further tools to develop mechanistic hypotheses and connect chemical profiles with putative biological targets [18], which complements experimental findings especially when antibiotic synergy is observed.

Finally, toxicological studies and clinical case reports have brought to light harmful effects related to the traditional use of *T. polium*, such as hepatotoxicity caused by some neo-clerodane diterpenes [19,20,21]. These findings highlight the need for a linked approach to safety evaluation. In this respect, in silico ADMET prediction can serve as a valuable tool to forecast the pharmacokinetic profile and potential toxicological risk of leading contents [22,23].

In this context, it is of interest to valorize the therapeutic effects of *T. polium* in a large scale survey that encompasses GC–MS-based phytochemical characterization of its EO and phenolic extracts quantified by HPLC/UV–ESI–MS, determination of antioxidant activity with complementary methods (DPPH, FRAP, and CAT), determination of antimicrobial potency against MDR clinical strains, a study on interactions between EO and antibiotics through a synergy approach (FICI index), and an investigation based on molecular docking-based screening aimed to determine the mechanism of interaction between major components and proteins involved in antibiotic resistance. Finally, ADMET analysis was carried out to forecast the pharmacokinetic and toxicological properties of the most active molecules.

## 2. Materials and Methods

### 2.1. Plant Material

**Figure 1:**
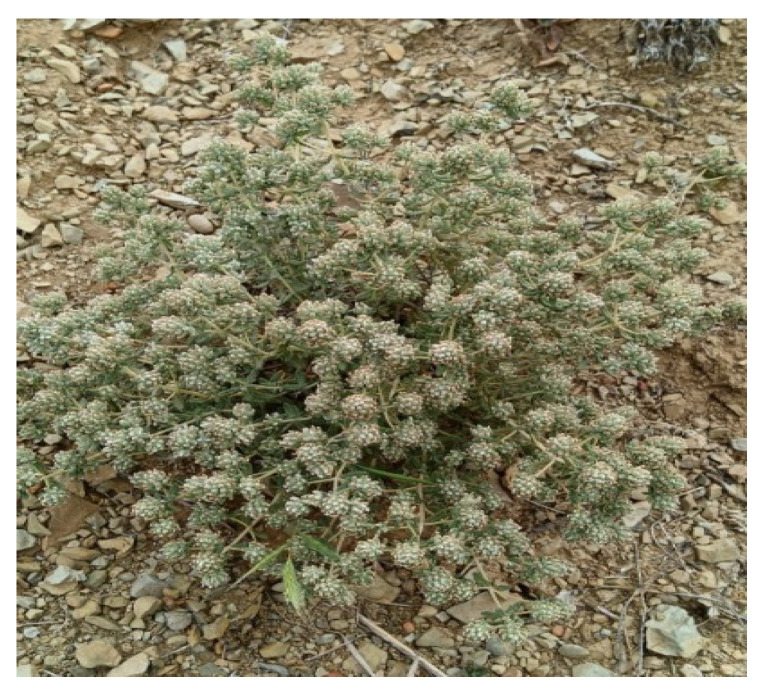
*Teucrium polium* (Zibouh Khalid and Touriya Zair, 2024).

This study investigated *T. polium* (*Lamiaceae*), a medicinal species collected in the Taza region (Northern Morocco) between May and June 2024 (Figure 1; Table 1). The aerial parts were harvested at the flowering stage and air-dried in the shade at ambient temperature (20–25 °C) for 15 days. Taxonomic identification was confirmed by the National Herbarium of the Scientific Institute of Rabat (Mohammed V University), Department of Botany, where a voucher specimen was deposited.

### 2.2. Quality Control of Plant Material

#### 2.2.1. pH Determination

The pH was determined by adding 20 mL of preheated distilled water to 4 g of the powdered plant material. The mixture was then filtered and allowed to cool. pH was measured using a benchtop pH meter (HANNA HI2211, HANNA Instruments, Villafranca Padovana, Italy) equipped with an STPURE electrode by immersing the electrode directly into an appropriate volume of the obtained filtrate [24].

#### 2.2.2. Mineral (Ash) and Organic Matter Contents

Organic matter content was calculated by the difference between the initial mass and the residual mass after calcination. Briefly, 5 g of the ground sample was placed in a muffle furnace at 550 °C until a uniform light-gray or whitish residue was obtained, indicating complete combustion of carbonaceous material [25]. Organic matter (*OM*) and mineral matter (MM, ash) were calculated as follows:

(1) $$OM\%=\left(\;\frac{W_{0}-W_{1}}{TS}\right)*100$$

(2) MM%=100−OM%

OM%: organic material; W_0_: capsule and sample weight before calcination; W_1_: capsule and sample weight after calcination; TS: test sample; MM%: ash content.

#### 2.2.3. Moisture Content

Moisture content was determined according to the AFNOR standard (NF V03-402, 1985) [26]. Five grams of the sample was dried in a ventilated oven at 100 ± 5 °C for 24 h. The analysis was performed in triplicate, and the mean value was reported.

(3) $$WC\%=\frac{\left(m_{0}-m_{1}\right)}{m_{0}}*100$$

WC: water content (%); m_0_: mass before drying (g); m_1_: mass after drying (g).

#### 2.2.4. Titratable Acidity

Powdered plant (5 g) was suspended in 50 mL of previously boiled distilled water. The resultant mixture was stirred for 15 min, and then, it was filtered. Then, 10 mL of the filtrate was added to 20 mL of distilled water, and after that, a few drops of phenolphthalein as an indicator were added. The resulting solution was titrated with 0.01 N NaOH until a faint persistent pink color is developed. Then, the volume of NaOH used was calculated and expressed as equivalents of citric acid [27]. Titratable acidity (TA) was measured according to:

(4) $$TA=\frac{weight\;of\;acid\;equiv.\;*normality\;of\;NaOH*vol.\;of\;titration\;\left(mL\right)*dilution\;factor}{sample\;weight\;(g)}$$

#### 2.2.5. Determination of Mineral Composition by ICP–AES

An inductively coupled plasma atomic emission spectrometry (ICP–AES) instrument (Agilent 5110, Agilent Technologies, Santa Clara, CA, USA) was used to quantify major and trace elements, including As, Cr, Sb, Pb, Cd, Fe, Cu, and Ti. The analytical procedure consisted of digesting 0.10 g of finely ground plant material with 3 mL of aqua regia (1 mL HNO_3_ + 2 mL HCl) under reflux at 200 °C for 2 h. After cooling, the digest was allowed to decant; the supernatant was collected and filtered through a 0.45 µm membrane. The filtrate was then brought to a final volume of 15 mL with distilled water prior to ICP–AES analysis [27,28].

### 2.3. Phytochemical Screening

The main chemical groups present in the investigated extracts were assessed by qualitative phytochemical screening. This approach is based on detecting characteristic color changes and/or precipitate formation following the addition of specific reagents. All tests were performed according to standardized and widely accepted phytochemical protocols [29,30].

### 2.4. Extraction and Characterization of T. polium EO

#### 2.4.1. EO Extraction and Yield Determination

EO was obtained from the flowering aerial parts by hydrodistillation using a Clevenger-type apparatus. Briefly, 100 g of plant material was boiled with 1 L of water for 3 h. The recovered EO was stored at 4 °C in a sealed amber glass vial until use. EO yield was calculated on a 100 g dry-matter basis using the following equation:

(5) $$Yield\;(\%)=\frac{V\;(EO)}{M_{0}}\times 100$$

V(EO): volume of EO recovered (mL); M0: mass of plant material (100 g).

#### 2.4.2. GC–MS Analysis and Identification of EO Constituents

EO analysis was performed on a gas chromatograph (HP 6890 series; Hewlett Packard, Palo Alto, CA, USA) equipped with a DB-5 capillary column (5% phenyl-methylsiloxane; 30 m × 0.25 mm i.d., 0.25 µm film thickness) and coupled to a mass spectrometer (HP 5973 series). A flame ionization detector (FID) was operated at 250 °C with an H_2_/air gas mixture. Ionization was carried out by electron impact at 70 eV. The oven temperature program was set from 50 to 200 °C at 4 °C/min, followed by a 5 min hold. Samples were injected in split mode (split ratio of 1:70). Nitrogen was used as the carrier gas at 1 mL/min. Instrument control and chromatogram acquisition were managed using HP ChemStation software version A.09.03.

Compound identification was based on the determination and comparison of Kovats retention indices (KIs) with reference values reported in Kovats (1965) [31], Adams (2017) [32], and Hübschmann (2015) [33]. Assignments were further supported by comparing retention times with available authentic standards and by matching KIs and mass spectra with those in the WILEY and NIST mass spectral libraries, as well as published data.

### 2.5. Extraction and Characterization of Phenolic Compounds

To isolate and characterize phenolic constituents from *T. polium*, two solid–liquid extraction procedures were employed. Soxhlet extraction using either distilled water or a hydroethanolic mixture (ethanol/water, 70:30, *v*/*v*), and traditional decoction. After extraction, the crude extracts were dried, collected in sterile vials, and stored at room temperature until further analysis.

### 2.6. Quantification of Phenolic Constituents

#### 2.6.1. Total Phenolic Content (TPC)

TPC was quantified by the Folin–Ciocalteu assay (Singleton et al. [34]), with minor modifications. A content of 20 µL of the extract (30 mg/mL) was mixed with Folin–Ciocalteu reagent (150 µL) and Na_2_CO_3_ solution (7.5%, 1.5 mL) in 50 mL graduated flasks and finally filled up to the mark with water. The homogenate was incubated in the dark for 45 min at room temperature. Absorbance was determined using 760 nm. Results were reported as GAE using a calibration curve that was prepared under the same conditions (y = 0.0738x − 0.0006; R^2^ = 0.998).

(6) $$TPC=\frac{C*V_{0}}{m_{extract}}*D$$

where TPC: total phenolic compounds; C: concentration assessed based on the calibration curve; V0: volume of the entire extract; m_extract_: mass of extract; and D: dilution factor.

#### 2.6.2. Total Flavonoid Content (TFC)

Total flavonoid content was estimated using the aluminum chloride (AlCl_3_) colorimetric assay [35]. In brief, 20 µL of extract was mixed with AlCl_3_ solution (10% *w*/*v*) and distilled water (2 mL). The final volume was brought to 5 mL with absolute methanol. The reaction mixtures were then kept in the dark at room temperature for 30 min after vigorous mixing. Absorbance was then measured at a wavelength of 433 nm. Concentration of flavonoids was reported as milligrams of quercetin equivalents per gram of extract (mg QE/g), with the use of a quercetin standard curve prepared within the range 0–30 µg/mL (y = 0.0579x − 0.0012; R^2^ = 0.999).

(7) $$TFC=\frac{C*V_{0}}{m_{extract}}*D$$

where FC: flavonoid content; C: concentration assessed based on the calibration curve; V0: volume of the entire extract; m_extract_: mass of extract; and D: dilution factor.

#### 2.6.3. Total Condensed Tannins (TCTs)

Condensed tannins were quantified using the vanillin–HCl assay [36]. Aliquots of 30 µL of each extract were mixed with 3 mL of vanillin solution (4% in methanol) and 1.5 mL of concentrated HCl (37%). After 20 min of incubation at room temperature, absorbance was measured at 499 nm. Quantification was performed using a catechin calibration curve (0–20 µg/mL; y = 0.041x + 0.0075; R^2^ = 0.996), and results are expressed as mg catechin equivalents per gram of extract (mg CE/g E).

(8) $$TCT=\frac{C*V_{0}}{m_{extract}}*D$$

where TCT: total condensed tannin; C: concentration assessed based on the calibration curve; V0: volume of the entire extract; m_extract_: mass of extract; and D: dilution factor.

#### 2.6.4. Identification of Chemical Composition by HPLC–ESI–MS

Compound identification was performed using ultra-performance liquid chromatography coupled to mass spectrometry (UPLC–MS) with a Dionex UltiMate 3000 system equipped with an Exactive mass spectrometer fitted with an electrospray ionization (ESI) source and an Orbitrap analyzer. A 10 µL aliquot of extract (100 µg/mL in distilled water) was injected onto a C18 column (100 mm × 2.1 mm; 1.7 µm particle size). The column temperature was set to 30 °C, and the flow rate was 0.45 mL/min.

The mobile phase consisted of solvent A (water with 0.1% formic acid) and solvent B (acetonitrile with 0.1% formic acid), delivered using the following gradient: 98% A/2% B (0–19 min), 70% A/30% B (20–24 min), 5% A/95% B (25 min) and then re-equilibration to 98% A/2% B (26–30 min). Detection combined a diode-array detector (UV scan 280–360 nm) and MS acquisition in negative ion mode. Data were processed using MassLynx software (v. 4.2). Compound assignment relied on retention times, mass spectra, molecular masses, and comparison with standards analyzed under identical conditions, including phenolic acids (gallic, caffeic, ferulic acids, etc.), flavonoids (e.g., rutin, catechin, and apigenin), and other polyphenols (e.g., tannic acid and coumarin).

### 2.7. Antioxidant Activities

Three complementary assays, DPPH, FRAP, and total antioxidant capacity (TAC/CAT), were used to evaluate the antioxidant potential of *T. polium* extracts.

#### 2.7.1. DPPH Radical Scavenging Assay

A DPPH• solution (6 × 10^−5^ M) was prepared by dissolving 2.4 mg of DPPH• in 100 mL of absolute ethanol. To assess antioxidant activity, variable volumes of each extract (at different concentrations) were transferred into test tubes and adjusted to a final volume of 200 µL with absolute ethanol. Subsequently, 2.8 mL of the DPPH• solution was added. Ascorbic acid served as the positive control, and a blank was prepared using absolute ethanol. Samples were incubated in the dark for 30 min, and the decrease in absorbance was measured at 515 nm against the negative control [37]. Results are expressed as the percentage of DPPH inhibition (AA%) using the following equation:

(9) $$AA\%=\frac{A_{control}-A_{sample}}{A_{control}}\times 100$$

AA%: percentage antioxidant activity; A_control_: absorbance of DPPH• solution without extract; A_sample_: absorbance of reaction mixture containing extract and DPPH•.

#### 2.7.2. Ferric Reducing Antioxidant Power (FRAP) Assay

The ferric reducing power of the extracts was evaluated based on the method described by Oyaizu (1986) [38]. The assay relies on the ability of antioxidant compounds in the samples to reduce ferric chloride. In brief, various concentrations of 0.5 mL of extract were combined with 2.5 mL of sodium phosphate buffer (0.2 M, pH 6.6) and 2.5 mL of 1% potassium ferricyanide. After 20 min of incubation at 50 °C, the reaction was stopped with addition of 2.5 mL of 10% trichloroacetic acid. The mixture was centrifuged at 3000 rpm for 10 min, and the supernatant (2.5 mL) obtained was mixed with distilled water (2.5 mL) along with ferric chloride solution (0.1% *v*/*v*) (0.5 mL). Absorbance was read at 700 nm, and the reducing activity was determined with ascorbic acid.

#### 2.7.3. Total Antioxidant Capacity (TAC)

Total antioxidant activity was determined according to the phosphomolybdenum method reported by Prieto et al. (1999) [39]. This method estimates the global reducing ability of the sample. In this assay, 10 µL of extract was mixed with 3 mL of a reagent solution containing sulfuric acid (0.6 M), sodium phosphate (28 mM), and ammonium molybdate (4 mM). The mixture was incubated at 95 °C for 90 min and then allowed to cool to room temperature before absorbance was measured at 695 nm. Antioxidant capacity was expressed as mg of ascorbic acid equivalents per g of extract (mg AAE/g E).

### 2.8. Antimicrobial Activity

#### 2.8.1. Microbial Material

The antimicrobial activity of the different extracts and the EO of the investigated plant was evaluated against five bacterial strains and five fungal strains. In addition, the synergistic effect of the EO in combination with selected conventional antibiotics was assessed against multidrug-resistant (MDR) clinical isolates (Table 2) recovered from Provincial Hospital Mohamed V (Meknès, Morocco). These microorganisms are recognized for their high pathogenicity, marked resistance to antibiotics, invasive potential, and clinical relevance, as they are frequently implicated in nosocomial infections in Morocco and represent a major public health concern. All strains were retrieved from stocks stored at −80 °C in 20% (*v*/*v*) glycerol, revived in Mueller–Hinton broth, and subcultured prior to testing.

#### 2.8.2. Antimicrobial Compounds

The eight antibiotics selected for this study were chosen because they represent distinct mechanisms of action and are widely used in hospitals to manage nosocomial infections caused by MDR bacteria. Antibiotics were purchased from Sigma-Aldrich (Darmstadt, Germany) and Acofarma (Barcelona, Spain). Details are provided in Table 3.

#### 2.8.3. Preparation of Antibiotics and Bacterial Inoculum

Antibiotics were dissolved in 0.9% NaCl solution. Bacterial strains were cultured on Mueller–Hinton agar (MHA). Each bacterial suspension was prepared by emulsifying 2–3 colonies from fresh MHA plates in 10 mL of distilled water to obtain turbidity equivalent to 0.5 McFarland.

#### 2.8.4. Determination of MIC, MBC, and MFC

Minimum inhibitory concentrations (MICs) of the different extracts were determined using the 96-well microdilution method in liquid medium [40,41]. A stock EO solution (50 mg/mL) was prepared in 10% DMSO, and extract stock solutions (100 mg/mL) were prepared in distilled water. Serial dilutions were performed in Brain Heart Infusion (BHI) broth for bacteria and Sabouraud medium for fungi to obtain a final volume of 100 µL per well. A standardized microbial inoculum was then added to each well (10^6^ CFU/mL for bacteria and 10^4^ CFU/mL for fungi). After incubation for 24 h at 37 °C, 10 µL of triphenyltetrazolium chloride (TTC; 0.5 mg/mL) was added as a colorimetric growth indicator. Following an additional 2 h of incubation at 37 °C, MIC was defined as the lowest concentration of EO or extract that completely inhibited growth, as indicated by the absence of TTC reduction (no red color development). In each series, the 11th and 12th wells served as growth and sterility controls, respectively. All experiments were performed in duplicate. Solvent control wells containing the corresponding DMSO concentrations (matching the EO dilutions) were included to exclude solvent-related growth inhibition.

For the determination of minimum bactericidal concentration (MBC) and minimum fungicidal concentration (MFC), 10 µL aliquots from all wells showing no visible growth were inoculated onto Mueller–Hinton agar for bacterial strains and Sabouraud agar for fungal strains. After 24 h of incubation at 37 °C, MBC (or MFC) was defined as the lowest concentration producing a 99.99% reduction in microbial load relative to the growth control. The MBC/MIC (or MFC/MIC) ratio was calculated to classify the activity as bactericidal (ratio < 4) or bacteriostatic (ratio > 4) [42].

#### 2.8.5. Checkerboard Microdilution Assay for Combination Testing

Checkerboard microdilution assays were performed to assess interactions between the EO and antibiotics [5,43]. Serial dilutions of EO and each antibiotic were prepared, and different combinations were tested. Each well contained 100 µL of Mueller–Hinton broth with the EO–antibiotic mixture at defined concentrations, plus 100 µL of bacterial suspension, for a final volume of 200 µL. Plates were sealed with sterile plate sealers and incubated at 37 °C for 24 h. Interactions were quantified using the fractional inhibitory concentration index (FICI). For the combination of EO (A) and antibiotic (B), FICI was calculated as shown below [44]. Solvent control wells (DMSO at the highest final concentration used in EO-containing wells) were systematically included.

(10) $$FICI={FIC}_{A}+{FIC}_{B}=\frac{{MIC}_{A+B}}{{MIC}_{A}}+\frac{{MIC}_{B+A}}{{MIC}_{B}}$$

where FIC_A_ corresponds to the MIC of the EO in the presence of the antibiotic (MIC_A+B_) divided by the MIC of the EO alone (MIC_A_) and FIC_B_ corresponds to the MIC of the antibiotic in the presence of the EO (MIC_B+A_) divided by the MIC of the antibiotic alone (MIC_B_). Interpretation followed commonly used chequerboard cut-offs (Table 4).

### 2.9. Molecular Docking

Molecular docking was performed to explore, from a mechanistic perspective, the ability of the major constituents of *T. polium* EO to interact with structural determinants of resistance in the investigated clinical strains. Target selection was guided by their involvement in the resistance phenotypes observed (β-lactamases, RND-type efflux, and outer-membrane permeability), the availability of experimentally resolved structures (X-ray crystallography or cryo-EM) from the relevant species, and the presence of a well-defined functional site (co-crystallized substrate/inhibitor or a described catalytic pocket), enabling reproducible definition of the docking grid. The selected proteins and associated grid parameters are presented in Table 5.

Crystallographic structures were obtained from the RCSB PDB database (accessed on 20 September 2025) and processed with PyMOL and AutoDockTools. We then omitted water molecules, co-crystallized ligands, and irrelevant heteroatoms in the preparation step. Polar hydrogens and Gasteiger charges were included afterwards, and the final structures were transformed into PDBQT format. Ligands (SDF files) were imported, energy-minimized, and converted into PDBQT using Open Babel. Docking calculations were carried out with AutoDock Vina, defining for each target a grid centered on the active site or functional cavity. For downstream analysis, the best-ranked pose and its binding energy were retained. The accuracy of the docking procedure was verified by redocking the co-crystallized ligand (when available) into the corresponding binding site and evaluating the root-mean-square deviation (RMSD) between the crystallographic and redocked poses (acceptance criterion: RMSD ≤ 2.0 Å). Docked complexes were examined and illustrated in 2D/3D using Discovery Studio Visualizer 4.6 to describe key interactions (hydrogen bonds, hydrophobic contacts, and involvement of catalytic or recognition residues).

### 2.10. In Silico ADMET Profiling and Toxicity Prediction (ProTox-II)

In silico predictions were performed using SMILES (Simplified Molecular Input Line Entry System) structures of the five major constituents of *T. polium* EO (α-pinene, β-pinene, limonene, α-muurolol, and β-eudesmol), obtained from PubChem (accessed 5 October 2024).

SwissADME (accessed 6 October 2024) and pkCSM (accessed 8 October 2025) were used to predict physicochemical and pharmacokinetic properties (ADMET). SwissADME was additionally used to evaluate drug-likeness according to Lipinski’s Rule of Five. Toxicity prediction was conducted using the ProTox-II server (accessed 9 October 2024) [22]. Predicting these properties is critical to reducing the risk of late-stage failures during development [45].

### 2.11. Statistical Analysis

Quantitative phytochemical assays and antioxidant measurements were performed in triplicate and are expressed as means ± standard deviations (SDs). MIC and chequerboard assays were performed in duplicate and are reported as the modal values observed across replicates. Statistical analyses were carried out for triplicate quantitative datasets using GraphPad Prism v10.4.0 for Windows (GraphPad Software, San Diego, CA, USA). Comparisons among multiple groups were conducted using one-way analysis of variance (one-way ANOVA), followed by Tukey’s post hoc multiple-comparison test when appropriate. Statistical significance was set to *p* < 0.05.

## 3. Results and Discussion

### 3.1. Quality Control of T. polium Plant Material

Quality control of *T. polium* plant material was performed by monitoring moisture, pH, titratable acidity, and ash content. The summary of findings are presented in Table 6.

Physicochemical analysis reported a moisture level of 10.49%, which satisfies the WHO specification (≤12%) for proper storage and a low level of microbial spoilage [46]. The presented sample had a slightly acidic pH (5.46) accompanied by a relatively high titratable acidity content (10.25%), hence indicating an important contribution of organic acids and phenolic compounds. Ash content (8.92%) was still within an acceptable range (<10%). Taken together, these results are in agreement with those described by the European Pharmacopoeia [47].

### 3.2. Mineral Composition by ICP-AES

Elemental analysis of samples of *T. polium* flowering tops by ICP–AES to determine some trace elements (Fe and Cu) in addition to heavy metal contents (As, Pb and Cd) was performed, and the results are presented in Table 7.

Elemental contents are reported as mg/kg dry weight (Table 7) and were calculated from ICP–AES readings using (C_sample_ (mg/kg) = C_solution_ (mg/L) × V_final_ (L)/m_sample_ (kg)), based on the digestion mass (0.10 g) and final volume (15 mL).

Lead (Pb) and cadmium (Cd) were not detected within limits set by European Pharmacopoeia regulations (Pb ≤ 5 mg/kg; Cd ≤ 1 mg/kg) and the FAO/WHO [47]. The arsenic (As) level was still below the FAO/WHO value and the ICH Q3D maximum acceptance criterion for elemental impurities in herbal materials (1.5 mg/kg) [48]. The concentration of antimony (Sb) was below the limit reported by the FAO/WHO and that defined by ICH Q3D for oral exposure, but it is not targeted specifically in the European Pharmacopoeia [48]. The detected Cr concentration was far lower than in the FAO/WHO (2 mg/L) and ICH Q3D system values provided in the literature (1100 mg/kg) [19,47]. The metal titanium (Ti) was found at the very low concentration value of 0.0322 mg/L; it is not cited in the European Pharmacopoeia, ICH Q3D guidelines or FAO/WHO limits [19,47], but it can be due to soil condition and must be controlled during medicinal plant quality control according to WHO recommendations (WHO) [49]. Additionally, copper and iron physiologically relevant trace elements were measured. The iron level was observed, and it was below the FAO/WHO limit, indicating possible accumulation due to soil characteristics and/or cultivation conditions [50,51].

### 3.3. Phytochemical Screening

Phytochemical screening is an essential preliminary step, as it highlights the major families of constituents associated with biological activities. The results obtained for *T. polium* flowering tops (Table 8) reveal a wealth of primary and secondary metabolites. Indeed, our plant is rich in proteins, lipids (sterols and triterpenes), carbohydrates (oses and holosides), and mucilage, as well as a marked set of polyphenols, particularly flavonoids (anthocyanins and flavones) and tannins. Saponins are also present. However, no alkaloids (tested using Mayer, Dragendorff, and Wagner reactions) or leucoanthocyanins were detected. The abundance of polyphenols is consistent with the findings obtained by Sharifi-Rad et al., who showed that the flowering stage corresponds to high levels in *T. polium* [52]. Furthermore, these results are consistent with the literature, as many researchers have confirmed the presence of primary metabolites, polyphenols, and flavonoids in *T. polium* [53,54].

### 3.4. Diversity of Volatile Constituents in T. polium

#### 3.4.1. EO Yield and Quality Control

Quality control of *T. polium* EO indicated a density of 0.931 g/mL at 20 °C and an organoleptic profile characterized by a dark-yellow color and a strong, pronounced aroma (Table 9). This density falls within the ranges reported for several *Lamiaceae* species described in the European Pharmacopoeia.

The EO yield was 0.72% (Table 9), which lies within the range reported for *T. polium* (0.20–1.65%), depending on geographic origin and phenological stage [55,56]. This yield is higher than that reported in Algeria by Belmekki et al. (0.21%) [57] and lower than the highest value described in Saudi Arabia (1.65%) [58]. Comparable yields were also reported by El Atki et al. for two Moroccan subspecies of *T. polium* (0.90% and 0.75%) [10].

#### 3.4.2. GC–MS Chemical Profiling of *T. polium* EO

**Figure 2:**
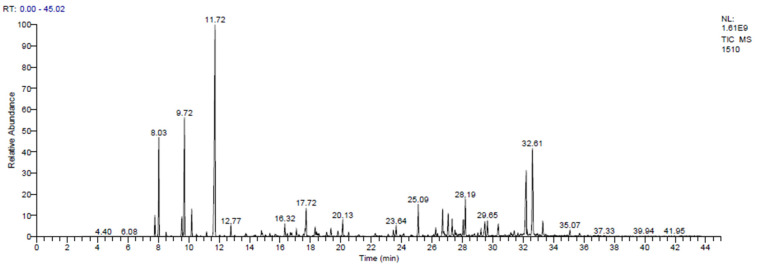
Normalized GC–MS chromatographic profile of the studied *T. polium* EO.

**Figure 3:**
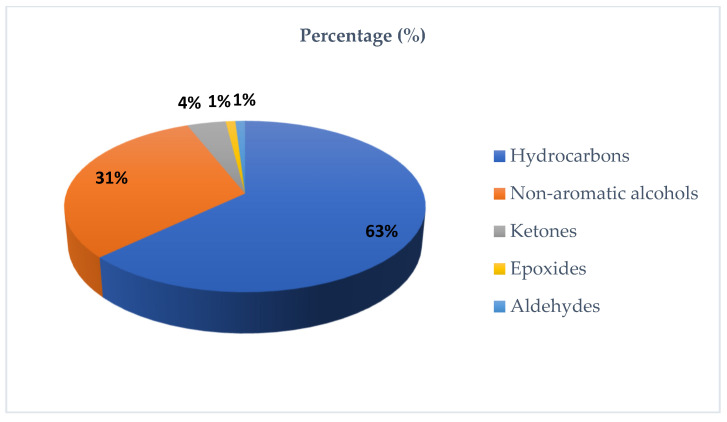
Distribution of the main chemical classes in *T. polium* EO.

The chromatogram obtained from GC-MS analysis of the EO extracted from the flowering tops of *T. polium* is shown in Figure 2. The percentages of metabolite classes identified for this species are described in Figure 3.

The chemical composition of the EO is presented in Table 10. The GC–MS profile was dominated by monoterpene hydrocarbons (48.60%) and oxygenated sesquiterpenes (28.36%), followed by sesquiterpene hydrocarbons (13.94%) and oxygenated monoterpenes (9.11%). The major constituents were limonene (24.13%), β-pinene (10.10%), α-pinene (7.89%), β-eudesmol (10.48%), and α-muurolol (8.10%). Additional notable compounds included (E)-caryophyllene (2.84%), caryophyllene oxide (1.25%), and (E)-nerolidol (1.35%). Limonene is widely documented for anti-inflammatory and antimicrobial activities [59]. Pinene isomers have been associated with antibacterial and anti-inflammatory effects and have also been discussed as potential modulators of antibiotic resistance, particularly in EOs from conifers and Lamiaceae [60]. Oxygenated sesquiterpenes such as β-eudesmol and α-muurolol have shown anti-inflammatory, neuro-/cytoprotective, and antibacterial signals in recent experimental models [61]. In addition, caryophyllene oxide is frequently implicated in the antifungal activity of EOs, whereas nerolidol has been reported to inhibit bacterial biofilm formation [62].

The EOs from *Teucrium* species are mainly formed by sesquiterpenes (germacrene D and β-caryophyllene) and also some monoterpenes (α- and β-pinene) [7]. In contrast, volatile oils extracted from a cohort of Iranian plants (Kashan region) showed α-pinene as the principal compound (9.67%), followed by β-caryophyllene (8.07%), β-pinene (5.04%) and nerolidol (4.94%), providing an alternate terpene profile [63]. On the other hand, the abundance of α-humulene (19.99%), β-pinene (16.94%) and spathulenol (8.40%) in EO harvested from eastern Algeria indicates a rather sesquiterpene hydrocarbon-dominated chemotype, with respect to the current one, which is more related to limonene and oxygenated sesquiterpenes [64]. In contrast, a similar profile to that in our study was other- recorded in Algeria and was dominated by monoterpene hydrocarbons with limonene (34.7%), α-pinene (25.4%) and β-pinene (8.6%) [65]. In Morocco, Benali et al. reported a chemotype mainly characterized by β-pinene (19.82%), germacrene D (18.33%), α-cadinol (6.83%) and limonene (5.71%), and El Atki et al. described separate profiles according to the subspecies, supporting that site, climate and time of harvest greatly influence chemodiversity [10,66]. This intra-specific and geographic variability may possibly be responsible for the different biological activities, with various potential applications which could explain the wide range of traditional uses described for *T. polium* EO.

### 3.5. Extraction Yields and Contents of Total Polyphenols, Flavonoids, and Condensed Tannins

Extraction yields as well as the total amounts of polyphenols, flavonoids, and tannins were evaluated in three sets of extracts from *T. polium* (decoction, aqueous extract, and hydroethanolic extract). The variation was found to be significant according to both the extraction solvent and the procedure (Table 11).

The extraction recovery was greatest in the aqueous extract (23.91 ± 0.58%), followed by the decoction (20.98 ± 0.55%) and hydroethanolic extract (20.56 ± 0.99%). This observation is in agreement with studies that indicated water as the best extraction solvent in many cases, primarily because of co-extraction of highly polar constituents [67]. Nevertheless, the level of phenolic metabolites in the aqueous extract was lower than the hydroethanolic one even though it had a higher yield. In fact, the hydroethanolic extract showed the highest total polyphenol (196.21 ± 1.92 mg GAE/g extract) and flavonoid (21.44 ± 0.38 mg QE/g extract) contents, surpassing significantly both the decoction (144.72 ± 6.70 mg GAE/g and 9.82 ± 0.08 mg QE/g, respectively) and the aqueous extract (139.97 ± 0.96 mg GAE/g and 11.94 ± 0.30 mg QE/g). These results also verify the efficacy of water/ethanol mixtures, which have intermediate polarity, allowing for the solubilization of a wider range of phenolic constitutions.

In the case of catechin-type tannins, the opposite occurred, with the decoction being richer (12.31 ± 0.23 mg CE/g extract), followed by the aqueous extract (11.52 ± 0.22 mg CE/g extract); finally, the hydroethanolic extract presented a lower amount (10.10 ± 0.03 mg CE/g). This implies that the action of heat (warming) in the decoction could improve the extraction of condensed tannins; however, some other flavonoids (quite often more thermolabile entities) might also undergo partial degradation after prolonged heating.

In general, these results are in agreement with previous reports. El Atki et al. observed that the polyphenol and flavonoid contents in hydroalcoholic extract from *T. polium* were significantly higher than those in aqueous extracts and proposed the mixed solvent system to be an efficient extractant for phenolic plant material [8]. The work by Antony et al. and that of Pizzi et al. reported that hot extraction methods such as decoction can promote the recovery of condensed tannins, whilst other thermosensitive flavonoids may suffer degradation under heating [68,69].

On one hand, the overall polyphenol contents reported in the present study tend to be at least on the higher limit of previously published values for *T. polium*, depending on solvent system and extraction protocol [8,70]. A previous work described an ethanolic extract which contained 227.43 mg GAE/g and 20.78 mg QE/g, similar to the values obtained in this work for flavonoids but with a slightly higher concentration of polyphenols, confirming the efficacy of hydroalcoholic solvents in extracting these metabolites [54]. Regarding the aqueous extract, Ait Chaouche et al. recorded superior TPC (184.84 mg GAE/g extract) and TFC (38.95 mg QE/g extract) compared with what was obtained here, which could be a result of chemotype, climatic conditions, geographical location and the time of harvesting [70].

### 3.6. Analysis and Identification of Polyphenols in T. polium Extracts by HPLC/UV–ESI–MS

**Figure 4:**
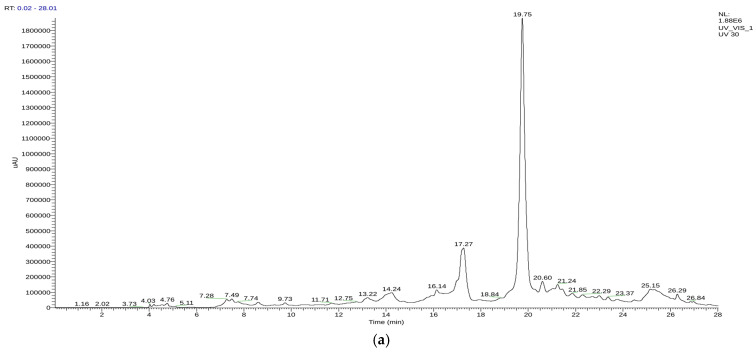

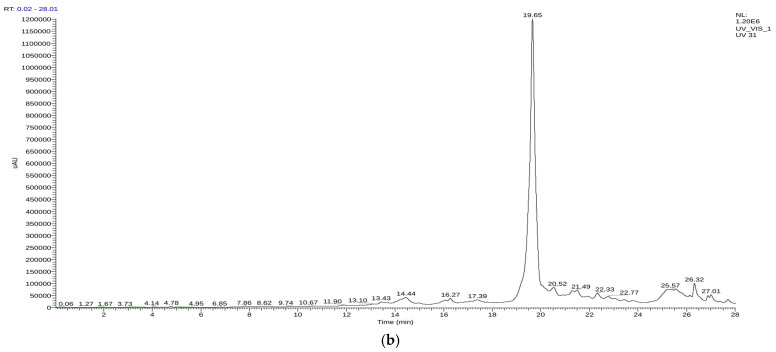
HPLC/UV–ESI–MS chromatograms of *T. polium* extracts: (**a**) decoction; (**b**) hydroethanolic extract.

The *T. polium* extracts were then examined with liquid chromatography–mass spectrometry (HPLC/UV–ESI–MS) in negative ionization mode. The corresponding chromatographic profiles are shown in Figure 4.

The annotation using HPLC/UV–ESI–MS discovered a wide spectrum of secondary metabolites in the hydroethanolic extract and decoction. In general, the identified compounds were predominantly flavonoids (53%), phenolic acids (19%), other polyphenolic compounds (13%), tannins (4%), terpenoids (6%) and hydroxycinnamic amides (2%). This predominance of phenolic compounds certifies the high polyphenol content in *T. polium*, which is known for its abundant flavones and phenolic acids. The latter results are in line with those obtained by Özer et al. (2017) [71] and Chabane et al. (2021) [72] indicating that phenolic acids are overwhelmingly responsible for providing the intrinsic antioxidant capacity of such species, as suggested by Moghadam et al. (2022) [73].

Comparison of the two extracts showed distinct qualitative and quantitative differences. The decoction was particularly abundant in phenolic acids (caffeic, cinnamic, quinic, chlorogenic, diferulic, and rosmarinic acids), as well as flavonoids such as myricetin, catechin 3-*O*-rhamnoside luteolin and apigenin.

In contrast, the hydroethanolic extract had higher contents of glycosylated flavonoids (e.g., quercetin 3-*O*-glucoside and luteolin 7-*O*-rutinoside) and some typical polyphenols, such as teucardoside, epigallocatechin gallate, and acteoside, due to the solubilizing properties of the water/ethanol system. This distributions is consistent with the results obtained by Özer et al. (2017) [71], who reported that solvent polarity has a strong impact on extract composition: decoctions usually show higher levels of free phenolic acids, whilst glycosylated flavonoids are readily extracted from ethanolic systems. Concordant results were demonstrated by Noumi et al. (2020) [74], highlighting the importance of the choice of solvent for the recovery of different polyphenolic profiles.

With respect to the major compounds, epicatechin was most abundant in the decoction (38.63%), followed by catechin 3-*O*-rhamnoside (12.11%) and naringenin 7-*O*-glucosid (2.88%), suggesting that the majority of flavonoids were catechin derivatives. Hydroethanolic extract also showed significant contents of epicatechin (56.91%), quercetin 3-*O*-glucoside (3.38%), luteolin (4.61%) and apigenin (4.37%). Epicatechin, luteolin and apigenin were detected in both extracts. These findings are consistent with Moghadam et al. (2022) [73], who pointed out the common predominance of these flavonoids using diverse extraction methods and indicated their importance as potential chemical markers for this species.

The fact that epicatechin, luteolin, and apigenin were uniformly identified in all extracts supports their evaluation as specific components to be considered chemical markers of *T. polium*. The complete annotated compounds and their relative levels are listed in Table 12, which demonstrates the differences between the decoction and hydroethanolic extract in terms of phenolic and flavonoid composition in a solvent-dependent manner.

### 3.7. Antioxidant Activity of EO and Extracts of T. polium

The antioxidant potential of *T. polium* preparations was assessed using three complementary assays: DPPH radical scavenging, total antioxidant capacity (TAC), and ferric reducing antioxidant power (FRAP). The results are summarized in Table 13.

#### 3.7.1. Total Antioxidant Capacity (TAC)

**Figure 5:**
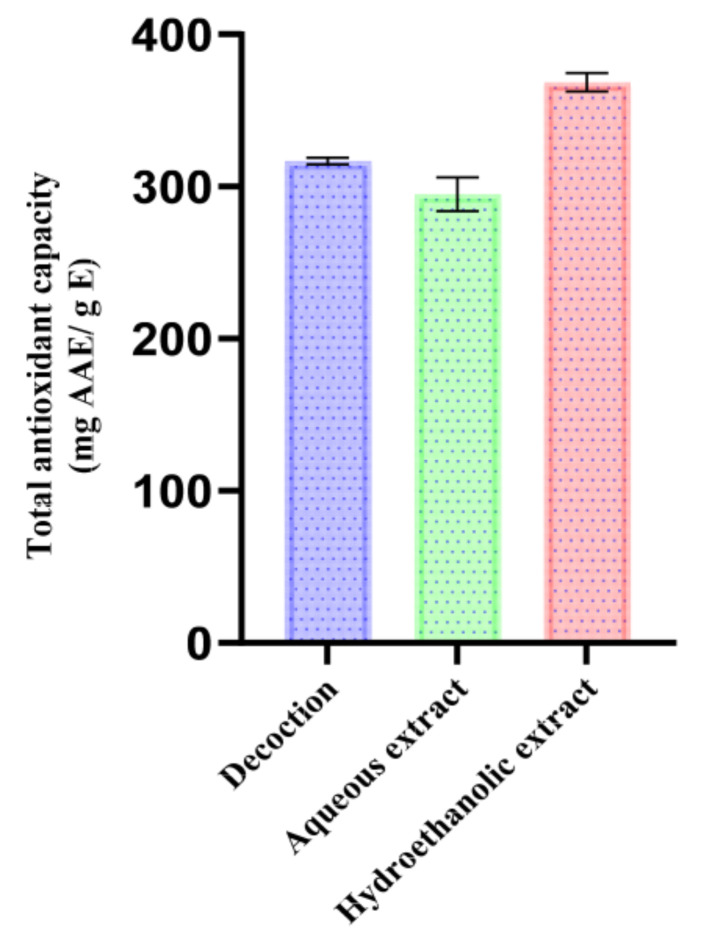
Total antioxidant capacity (TAC) of *T. polium* extracts.

The total antioxidant potential of aqueous and hydroethanolic extracts of *T. polium* according to the ammonium molybdate (phosphomolybdenum) method (mg AAE/g) is given in Figure 5. The method is based on the reduction of Mo (VI) to Mo (V) under acidic conditions, offering an overall estimation of the global reducing potential of extracts and thus demonstrating the cumulative influence of reducing agents like phenolics, as well as other primary/secondary metabolites.

The highest TAC value was recorded with the hydroethanolic extract (368.47 ± 6.14 mg AAE/g), followed by the decoction (316.60 ± 2.15 mg AAE/g) and the aqueous extract (294.79 ± 11.03 mg AAE/g). This ranking provides insights about the solubility of the extract and the efficiency of extraction, indicating a strong effect of the solvent system as well as the extraction condition. The hydroethanolic combination is a mid-level-polarity system that favors enhanced extraction of a high number of high-reducing-power constituents and, in our case, one which correlates with the highest total phenolics and flavonoids with at least a measurable catechin-type tannin fraction. Epicatechin (56.91%), luteolin (4.61%) and apigenin (4.37%) were the major metabolites determined in this extract, which could potentially strongly contribute to its redox activity.

Although total phenolic and flavonoid contents were slightly lower, the aqueous decoction showed an intermediate TAC presumably due to easily extracted water-soluble antioxidants such as chlorogenic acid (4.32%), luteolin (4.08%), and epicatechin (38.63%). On the contrary, the Soxhlet aqueous extract revealed the lowest TAC value; it might be attributed to partial decomposition of thermolabile phenolics and flavonoids on long heating exposure in hot solvent.

The Tanzanian *Polium* sample from the Errachidia region reported in the literature gave a higher TAC value for the aqueous extract (129.5 ± 3.19 mg AAE/g), followed by methanol (100 mg AAE/g) and ethanol (80 mg AAE/g) extracts, while ethyl acetate extract resulted in the least active one (21.70 ± 2.20 mg AAE/g) [75]. This solvent-driven trend is mostly due to the polarity effects (water drawing highly hydrophilic compounds; methanol removing polar or semi-polar molecules; ethanol favoring semi- rather than low-polarity fractions). Discrepancies from our results could be due to extraction temperature and protocol, as hot extraction can improve polar and semi-polar polyphenols release, as well ecogeographic variation (Errachidia: arid climatic zone; Taza: more temperate/humid), which could modulate secondary metabolism, hence apparent solvent hierarchy.

#### 3.7.2. DPPH Radical Scavenging Activity

Free radical scavenging activity was first evaluated by the DPPH assay, which determines the free radical scavenging ability through the reduction of the stable radical 2,2-diphenyl-1-picrylhydrazyl. EO of *T. polium* had mid-range activity, with an IC_50_ (24.06 ± 0.09 mg/mL) that was significantly lower when compared with the phenolic-rich extracts. This finding agrees with the profile of chemical composition of the EO, whose main component was monoterpene hydrocarbons (48.6%), such as limonene (24.13%), β-pinene (10.10%) and α-pinene (7.89%), which normally have low direct antioxidant capacity. Oxygenated sesquiterpenes (28.36%), on the other hand, are possibly mostly responsible for the activity observed, with that of α- and β-eudesmol being 10.48%, that of α-muurolol being 8.10% and that of caryophyllene oxide being 1.25%, as they have oxygenated groups that may aid radical-quenching processes. Oxygenated monoterpenes (terpinen-4-ol, 2.72%; carvone, 1.39%) may also make a less pronounced contribution. Considering the high content of oxygenated compounds in the oil, its moderate antioxidant capacity probably arises from their synergy but is lower than that of phenolic-containing extracts and confirms the primary contribution of polyphenols to radical scavenging.

**Figure 6:**
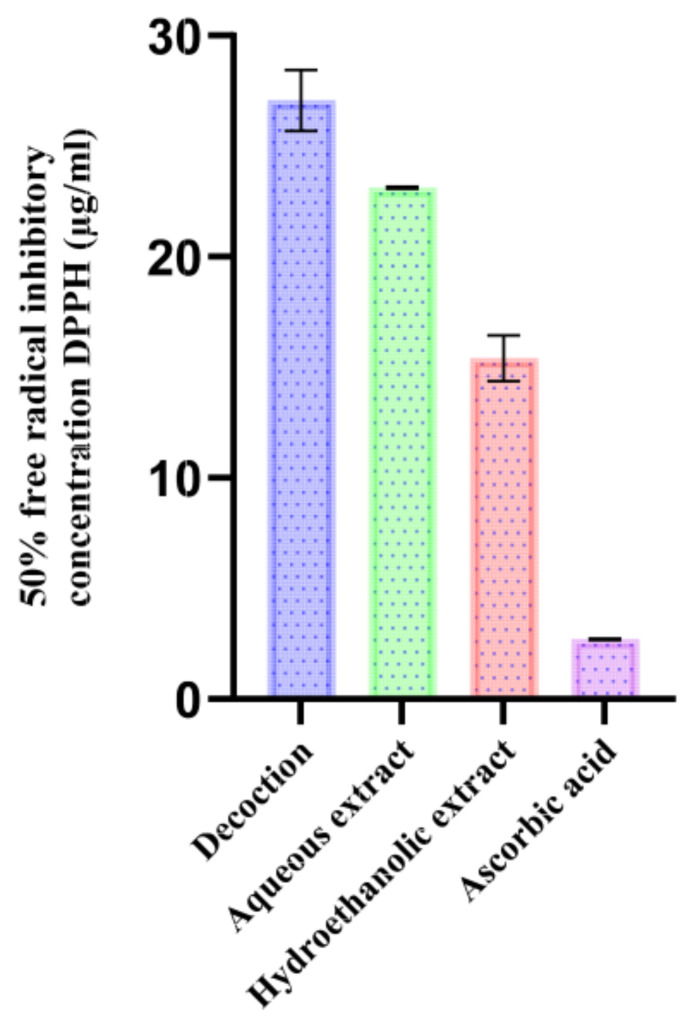
DPPH IC_50_ values of aqueous and hydroethanolic *T. polium* extracts.

Figure 6 shows the IC_50_ DPPH values of the various extracts from *T. polium*. Significant differences among the extraction methods were observed for all extracts in terms of their antioxidant capacities (DPPH scavenging activity), indicating that both the solvent and operating conditions had a marked impact on antioxidant efficiency. Ascorbic acid was used as the standard.

The hydroethanolic extract showed the most potent activity (IC_50_ = 15.41 ± 1.04 µg/mL), followed by the aqueous extract (IC_50_ = 23.12 ± 0.04 µg/mL) and the decoction (IC_50_ = 27.07 ± 1.38 µg/mL). This gradient would support the higher recovery of antioxidant metabolites under the hydroethanolic system, with its intermediate polarity that allows for the co-extraction of different phenolic classes. The relatively weaker activity of aqueous extracts suggests that some antiradical compounds may require optimal contact time for efficient extraction; for example, prolonged hot extraction could also lead to partial destruction of thermosensitive components.

Furthermore, flavonoids have been identified as potent antioxidants [76] and seem to be the major cause of the observed differences between the extracts. The decoction had 77.83% flavonoids, while cell wall-bound flavonoids were 89.111%; this challenges the hydroethanolic extract as far as total indicators of flavonoid content are concerned. That the ratio between aglycone and glucosides seems to play a central role is also indicated by the fact that the hydroethanolic extract shows a higher portion of aglycones (78.5%) than the decoction (55.89%), while the decoction is richer in glycosylated flavonoids (21.94%) than the hydroethanolic extract (10.61%).

Of the identified flavonoids, apigenin and rhamnetin (which are unique to the hydroethanolic extract) combined account for 5.4%, while cirsiliol concentration is 2.35-fold higher than in the decoction. These three flavones are known for their antioxidant activity [77,78,79,80] and may contribute to the much higher effectiveness of the hydroethanolic extract. On the other hand, apigenin glycosides, amounting to 3.87% in the decoction, are usually reported to be less active in comparison with their corresponding aglycones. This explanation is supported by a comparative study which demonstrated that glycosylation decreases in vitro antioxidant activity [81], which would be in keeping with the mechanism proposed.

Catechin-type compounds are also responsible for the high activity of the hydroethanolic extract, as demonstrated by Boulmokh et al. [82]. An in vitro standard, the reference radical trapper epigallocatechin gallate (1.58%), was found in this extract.

Also, epicatechin, another known antioxidant, was found to be in greater percentage abundance in the hydroethanolic extract (56.91%) than in the decoction (38.63%) [83]. This profile of phytochemicals, characterized by high content in aglycones and catechins with high reducing power, can justify the excellent antiradical activity of the hydroethanolic extract.

When compared with the available literature (Table 14), extracts from most regions are less active. For instance, Soxhlet extracts with 100% ethanol extracted in Algeria showed lower antiradical power (PAR of 49.23 and 50.58), meaning that possibly purely organic solvents retrieved a narrower focused fraction of polar antioxidants [54,84]. Regional variations could also be due to pedoclimatic factors (e.g., altitude, temperature, and solar exposure), which regulate polyphenol biosynthesis and consequently extract composition.

Similarly, Serbian samples macerated in water or methanol presented PAR of 70.92 ± 1.92 and 67.37 ± 0.86, respectively [85], indicative of moderate activity, which is expected when using solvents that are possibly less apt to simultaneously extract polar and semi-polar antioxidants.

Overall, the present extracts from *T. polium*, especially the hydroethanolic combination (7:3), was one of the most active extracts reported, meaning not only that phenolic content was abundant in the plant material but also that intermediate-polarity solvents are appropriate for the recovery of antioxidant potential.

#### 3.7.3. Ferric Reducing Antioxidant Power (FRAP)

**Figure 7:**
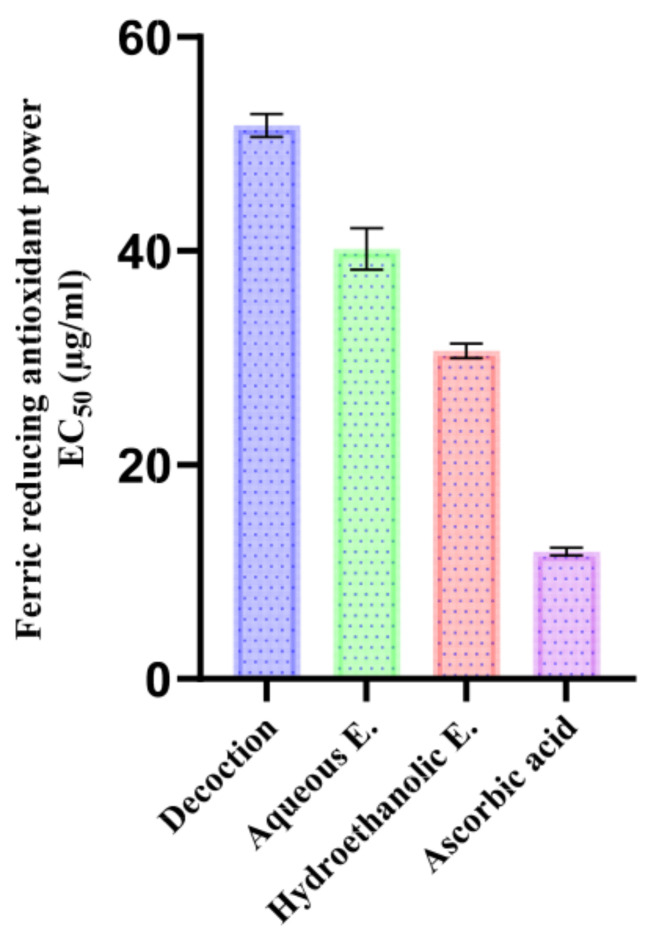
FRAP EC_50_ values of aqueous and hydroethanolic *T. polium* extracts.

FRAP results confirmed this trend (Figure 7), meaning that the hydroethanolic extract had a lower EC_50_ (30.65 ± 0.69 µg/mL), followed by the aqueous extract (40.18 ± 1.95 µg/mL) and the decoction (51.72 ± 1.08 µg/mL). This order might be justified by the presence of a higher content of flavan-3-ols (mainly epicatechin) and aglycone flavones/flavanols (luteolin and apigenin) in the EtOH/H_2_O extract exhibiting high efficiency in donating electrons, thus reducing the Fe^3+^ complex into Fe^2+^ in the FRAP system [86]. In contrast, simple phenolic acids are still reducing agents but, as a rule, less strong than polyhydroxylated flavonoids, which can account for the lower FRAP response of the decoction, which is richer in simpler phenolics and glycosyl forms [87].

In comparison with published research, El Atki et al. found EC_50_ of 395 µg/mL (ethanolic extract) and 456 µg/mL (aqueous extract), whereas El-Guourrami et al. studied a decoction; these values are significantly higher as compared with those obtained in the present study (lower activity) and point out the impact of solvent, extraction method, and chemotype on antioxidant activity optimization [8,88].

### 3.8. Antimicrobial Properties of T. polium EO and Extracts

#### 3.8.1. Susceptibility of Microbial Strains to Antibiotics and Antifungals

The susceptibility of a microorganism to one or more antibacterial or antifungal agents is ascertained with antibiograms and antifungalgrams. Bacterial susceptibility testing was performed with the automated BD Phoenix or VITEK 2 instrument, and fungal susceptibility was tested on microdilution plates. The MICs (µg/mL) are listed in Table 15 and Table 16.

#### 3.8.2. Antimicrobial Activity of EO and Extracts of *T. polium*

The MIC, MBC, and MFC values of *T. polium* EO and extracts are presented in Table 17.

In general, the EO of *T. polium* was found to be more potent than the extracts against all examined bacteria and fungi, especially in *Enterobacteriaceae* (*E. cloacae*, *K. pneumoniae* and *E. coli*). Regarding these Gram-negative species, the MIC and MBC of the EO were the same (25 mg/mL), indicating a bactericidal effect, whereas the hydroethanolic extract and the decoction in general showed higher MICs (50 mg/mL), and the aqueous extract remained ineffective at >50 mg/mL. In addition, S. aureus was more sensitive to the decoction (MIC = 25 mg/mL) than to EO (MIC = 50 mg/mL), and *S. epidermidis* kept on being inhibited by an EO MIC value equaling 25 mg/mL. *E. cloacae* was particularly resistant to some of the antibiotics used (gentamicin, amoxicillin–clavulanate, and trimethoprim–sulfamethoxazole), which the EO effectively inhibited. This finding is consistent with the hypothesis that EO has targets other than classical β-lactam and antifolate targets, which should be mainly represented by membrane-active effects inducing ionic homeostasis imbalance [89].

This activity is in keeping with the lipophilic constitution of EO, a mixture of monoterpenes (limonene and pinenes) and oxygenated sesquiterpenes (β-eudesmol), which are known to increase membrane permeability, disrupt transmembrane gradients, and also modulate virulence-connected traits, including in multidrug-resistant phenotypes [90,91]. On the other hand, extracts rich in phenolics (aglycone flavonoids, phenolic acids and tannins) are mainly related to efflux pump inhibition, metal chelation and interference with enzymes. These mechanisms may confer increased activity against Gram-positives (interaction with peptidoglycan and teichoic acids), yet they are usually thwarted by their outer-membrane permeability barrier in Gram-negatives [92,93]. Regarding fungi, compared with the extracts, the EO was more active against *Candida* spp., with MICs of 4.69–9.38 mg/mL, and *A. niger*, with 1.17 mg/mL. On the other hand, extracts showed MIC values ranging from 6.25 to 50 mg/mL. The three extracts showed the highest activity against *C. tropicalis* (MIC = 6.25 mg/mL). The hydroethanolic and aqueous extracts had the same MIC (6.25 mg/mL) against *C. dubliniensis*, and only moderate activity against *A. niger* (MIC = 50 mg/mL).

The antifungal effects of EO are ascribed to the presence of monoterpenes and other components, which can block ergosterol biosynthesis and PM-ATPase activity, causing the loss of cell membrane integrity in *Candida* spp. The presence is consistent with a membrane/ergosterol-based pharmacodynamic profile, with the high sensitivity of *A. niger* reported here fulfilling that criterion. Moreover, β-eudesmol has been reported to possess antimicrobial and anti-inflammatory effects, which may synergize the overall activity of EO [94,95,96]. Compared with the literature, our results are in harmony with previous observations indicating that while *T. polium* EOs are more powerful when compared with extracts, such variation is species-specific. For example, Ben Othman and others (Tunisia) found that pinene-enriched EO (α and β) was the most efficient against Proteus mirabilis, *S. aureus* and *C. freundii* but not effective against *A. fumigatus* [16]. This response parallels our finding that EOs are overall superior to extracts but variable among species; differences in efficacy may be due to chemotype and methodological variation [16]. In Morocco, El Atki et al. noted significant EO activity (as the MIC) against nosocomial bacteria, particularly *S. aureus* and *A. baumannii* (MIC = 2.81 mg/mL), and lower activity against *P. aeruginosa* (MIC = 5.62 mg/mL), in agreement with our results of dependence on bacterial strains as well as on Gram type [10]. Regarding extracts, Alreshidi et al. demonstrated good activity against *A. baumannii* and *S. pyogenes*; moderate activity vs. *P. aeruginosa*, *P. mirabilis* and *E. cloacae* (MIC = 25 mg/mL); and varying responses to *S. aureus*. This is consistent with our observation that the hydroethanolic extract and the decoction were less active than the EO against Enterobacteriaceae but could present a better comparison against *S. aureus* [11]. With regard to antifungal activity, Alreshidi and colleagues reported low and moderate inhibition of *Candida* spp. and less pronounced effects against *Aspergillus* spp. with a methanolic extract, and this is in overall agreement with our extract data [11]. In contrast, the findings obtained by Ben Othman et al. are not in line with our highly potent inhibition of *A. niger*, which serves to underline interspecies variability and established EO composition-dependent fungal inhibitory activity [16].

#### 3.8.3. Synergistic Antimicrobial Activity of *T. polium* EO in Combination with Antibiotics

The fractional inhibitory concentration index (FICI) values of *T. polium* EO in combination with conventional antibiotics are presented in Table 18 and were calculated using the chequerboard assay. Among the 49 planned EO–antibiotic pairs, 25 combinations yielded interpretable FICI values (the remaining pairs were not evaluable because at least one MIC was outside the tested concentration range and is reported as “-”). Within the interpretable dataset, three interactions were synergistic (FICI ≤ 0.5), eleven were additive (0.5 < FICI ≤ 1), and eleven were indifferent (1 < FICI < 4).

The emergence of multidrug-resistant (MDR) bacterial pathogens has severely compromised the clinical efficacy of various classes of antibiotics. Therefore combination approaches based on EO and reference antibiotic pairings represent a promising strategy to improve antimicrobial effect and potentially avoid treatment failure [5,97]. Current study shows that *T. polium* EO had mainly synergistic effects when used against clinically relevant isolates resistant to several antibiotics. In particular, the best synergistic effects were noticed for *K. pneumoniae* with the use of amikacin (FICI = 0.3125), while for *E. coli*, a positive interaction can be observed, especially with ceftazidime (FICI = 0.1406) and ceftriaxone (FICI = 0.5156) treatments; very good synergy was also detected for *S. aureus* with ceftazidime (FICI = 0.039). Instead, additive or indifferent interactions were dominant in non-fermenters including *P. aeruginosa* and *A baumannii* and many additional antibiotic pairs. These results are of particular interest in a context of poor baseline antibiograms indicating high initial MICs for several antibiotic families.

Such results are in agreement with the synergistic mode of action frequently attributed to EO. *T. polium* EOs, high in monoterpenes (especially limonene and pinenes) and sesquiterpenes, have been described to interfere with bacterial membranes, to enhance the permeability of the envelope and improve the penetration of antibiotics into intracellular or periplasmic targets; in addition they may induce damage to macromolecules as DNA or proteins [91]. This membrane-active target accounts mechanistically for the observed synergy with amikacin against *K. pneumoniae*, given that by greater drug uptake to the 30S ribosomal target, MICs fall into the combination range. The same logic would also apply to E. coli with third-generation cephalosporins, as enhanced access of antimicrobial agents into the periplasmic space could improve their ability to interact with penicillin-binding proteins and thereby reduce MICs. Similarly, for the EO–ciprofloxacin pairing against E. cloacae, an additive effect was observed, which is compatible with combined permeabilization and reduced efflux, depending on the strain-specific resistance background.

Besides permeabilization, efflux pump activity is also inhibited by several EO components, which in turn promotes antibiotic retention within the cell and may be related to antibiotics that can be exported, such as fluoroquinolones. This mode may synergize membrane effects and participate in the lower MICs against ciprofloxacin, depending on the strain-specific resistance background [98]. Further to inhibition of biofilm formation, EO may act against both adhesion and QSS-related communication, with the eventual result being a reduction in phenotypic tolerance of bacteria to antibiotics [99].

The distribution of responses in different species and antibiotic classes probably mirrors diversity in cell-envelope structure and resistance determinants. *P. aeruginosa* and *A. baumannii* mainly displayed indifferent or additive effects, presumably due to their inherently low outer-membrane permeability and highly active efflux systems, which perhaps impede the effect of an EO that behaves as a moderate permeabilizer. By contrast, particularly for Enterobacteriaceae and S. aureus, the cumulative effects of permeabilization and efflux inhibition seem more often to be sufficient to yield reductions in antibiotic MICs (as seen with the above synergistic indices). In general, these findings are consistent with the increasing literature on the ability of EOs to augment antibiotic activity against both Gram-negative and Gram-positive bacteria, including MDR isolates, by multi-targeting mechanisms and mechanistic synergy.

Distressingly, however, a reduction in the MIC of a combination as measured in vitro will result in clinical benefit only if that combination’s MIC is below internationally defined susceptibility breakpoints [100]. Although the reduction in certain EO–antibiotic combinations was large in the current study, many combination MICs were high when compared with standard interpretive categories for the organisms tested.

From a translational perspective, the relatively high antibacterial MICs observed for the EO and extracts (often in the mg/mL range) suggest that direct systemic use of crude preparations is unlikely without formulation optimization. Because EO constituents are lipophilic and volatile, their bioavailability and local concentration at the infection site can be improved by encapsulation approaches such as nanoemulsions, liposomes, and lipid-based nanocarriers, which have been reported to enhance stability, control release, and antimicrobial efficacy of essential oils and to facilitate topical or localized delivery. Accordingly, future studies should prioritize standardized nanoformulations and toxicological assessments to evaluate whether the in vitro potentiation observed here can be achieved at clinically relevant exposures [101,102].

### 3.9. In Silico Molecular Docking of the Main Constituents of T. polium EO

Based on the synergistic activities observed between *T. polium* EO and antibiotics, an in silico study was conducted to evaluate the possible interactions between the main constituents of EO and the vital bacterial proteins responsible for resistance. These selected proteins included nine targets covering various resistance mechanisms. The binding energy for each ligand–protein complex is presented in Table 19, and the ratings are expressed in kcal/mol. The different interactions between the target proteins and the ligands with the highest binding affinity are described in Table 20.

In sum, the docking findings provide a mechanistically consistent model to interpret experimental synergy. Oxygenated sesquiterpenes including β-eudesmol and α-muurolol revealed the lowest binding energies (about −6.0 to −8.6 kcal/mol) for various resistance targets, like RND efflux pumps (e.g., AcrB and MexB), β-lactam hydrolyzing enzymes (e.g., CTX-M, AmpC, TEM, and BlaZ), major outer-membrane porin (OmpK36) and cell-wall target (PBP). The more apolar monoterpenes (compounds with weaker binding in general; e.g., α-pinene and β-pinene) showed weaker binding at AcrB/MexB (approximately −4.0 to −6.8 kcal/mol), while limonene had an intermediate profile with stronger affinity for AcrB/MexB and moderate interactions with periplasmic enzymes.

An obvious, relatively direct mechanistic connection is apparent for combinations that include antibiotics whose activity is heavily afflicted by efflux. Highly predicted binding affinity of β-eudesmol and α-muurolol against AcrB (2DHH) and MexB (2V50) might rationalize functional impeding to RND pumps, which may lead up the intracellular accumulation of antibiotics, i.e., fluoroquinolones. Both AcrB and MexB are known contributors to multidrug resistance in *Enterobacteriaceae* and *P. aeruginosa* [103,104]. This finding is compatible with the synergism observed with ciprofloxacin on *E. cloacae*, to which the susceptibility of this compound is highly reduced through AcrB activity. Maintenance of neutral or hydrophobic ligands in the pump binding pocket may hinder conformational dynamics and restrict efflux, an effect that is becoming more commonly reported for RND pump inhibitors, which augment antibiotic activity by restricting binding-pocket plasticity [105].

Concurrently, the nature of docking energies for oxygenated sesquiterpenes against class A and C β-lactamases (CTX-M-15/TEM-1/AmpC) and BlaZ implicates a second, congruent lever beyond membrane permeabilization: the partial co-inhibition of enzymatic hydrolysis by occluding the active site entrance or a nearby pocket, which could prevent substrate turnover. This hypothesis is consistent with the substantial reduction in MICs for ceftazidime and ceftriaxone in ESBL-positive E. coli and for ceftazidime against S. aureus observed. In such phenotypes, CTX-M and TEM enzymes predominate, and the structural and catalytic features of CTX-M-15 are well characterized [106], reinforcing the hypothesis that a ligand-dependent steric effect slowing hydrolysis is plausible.

Regarding permeability determinants, OmpK36 in *K. pneumoniae* (6RD3) is a key portal of entry for β-lactams. Possibly, the relatively high affinity of the oxygenated sesquiterpenes for OmpK36 is due to transient interactions, the induction of which may further favor conductive states resulting in increased periplasmic access of cephalosporins as a possible explanation (which has previously been described in relation with susceptibility to cephalosporins and β-lactam/β-lactamase inhibitor combinations) [107]. Lastly, the predicted binding to PBP (3OCL) is consistent with the idea that terpenes may modulate the periplasm during antibiotic exposure, such that they make it more likely that an antibiotic would come into close contact with a target; this might explain how cephalosporins are more potent in some strain contexts [108]. However, with good docking scores to MexB or medium affinity for targets like OXA-23 and AmvA, the experimental interaction profiles were mostly additive or indifferent in *P. aeruginosa* and *A. baumannii*. This result is not surprising because of the tightness of the envelope barrier, efflux redundancy and target accessibility typical for these non-fermenters, resulting in a functional impact that is reduced when permeabilization or single-target modulation are moderate [109,110].

According to the docking results, we chose the seven most appealing targets (1BLZ, 2V50, 3OCL, 4JF4, 4WZ4, 2DHH and 6RD3) to further analyze the interaction of three major EO components, including α-muurolol, β-eudesmol and limonene. In general, hydrophobic (alkyl/π-alkyl/π-σ) interactions were the predominant pattern for all complexes, although β-eudesmol also contacted hydrogen bonds in a number of complexes and possibly increased stabilization and specificity.

In all β-lactamase targets (e.g., 1BLZ and 4WZ4) and PBP (3OCL), α-muurolol and β-eudesmol were consistently docked into the catalytic pockets, which may interfere with substrate binding/entering to hydrolyze or enhance antibiotic efficacy. RND efflux pumps (2V50 and 2DHH) for both sesquiterpenes but less for limonene displayed strong docking in a hydrophobic binding pocket, suggesting the interference of efflux as one strategy to potentiate intracellular retention of antibiotics sensitive to efflux. Lastly, docking to OmpK36 porin (6RD3) suggested preferential stabilization at the pore entrance relevant with an effect on outer-membrane permeability.

Overall, these interaction profiles appear to converge towards a multi-target mechanism of action (efflux modulation, permeability effects and partial inhibition of the resistance enzymes/targets), which that may account for the synergistic trends seen in checkerboard assays.

### 3.10. Determination of ADMET Profile and Predicted Toxicity (ProTox-II)

Predictions generated using SwissADME and pkCSM indicate that the major constituents of *T. polium* EO display overall drug-like physicochemical and pharmacokinetic features, including compliance with Lipinski’s Rule, a bioavailability score of 0.55, moderate lipophilicity, and low-to-moderate aqueous solubility (Table 21).

Monoterpenes (α-/β-pinene and limonene) have very low TPSA values (0 Å^2^), in agreement with their primarily hydrophobic nature. All compounds exhibit high estimated Caco2 permeability. Nevertheless, predicted human gastrointestinal absorption is greater for oxygenated sesquiterpenes than for monoterpenoids. Considering the quality measures, limonene is the only compound predicted to be a P-glycoprotein (P-gp) substrate and would have high risk of intestinal efflux, while no prediction of P-gp inhibition is observed for any compound.

Predicted distribution volumes (logVDss ~ 0.40–0.69 L/kg) indicate moderate tissue distributions; for certain monoclonal cHL, they were less than that of plasma, which would imply limited penetration into the extravascular and/or intracellular space. Plasma protein binding seems to be more extensive for β-eudesmol considering its higher unbound fraction (Fu = 0.16). The estimated indices for brain distribution (logBB ~ 0.60–0.82) and CNS permeability (logPS ~ −2.37 to −1.86) point towards potential BBB passage, with low-to-moderate CNS penetration depending on the compound involved.

None of the compounds are anticipated to inhibit the major CYP isoforms tested (CYP1A2, CYP2C19, CYP2C9, CYP2D6 or CYP3A4). β-Eudesmol is the only predicted CYP3A4 substrate and may therefore be an important candidate for drug–drug interactions involving this enzyme.

For monoterpenes, predicted total clearance is low to moderate; however, it is high for sesquiterpenes. None of these compounds are expected to be substrates for renal OCT2, indicating that elimination via this transporter would not seem to play a prominent role.

Finally, the ProTox-II and pkCSM predictions suggest no severe safety concerns (Table 22); since all compounds are negative for AMES mutagenicity testing, hepatotoxicity is not predicted, and neither is inhibition for hERG I/II channels. But, limonene, α-muurolol and β-eudesmol are predicted to cause skin sensitization, indicating that care should be taken for dermal exposure. Based on the OECD acute toxicity class, most substances could be classified as class 5 (low acute toxicity), whereas β-eudesmol belongs to class 4, showing the minimal predicted oral LD_50_ (2000 mg/kg). These results indicate on the whole a favorable predicted safety profile, with special focus on potential skin sensitization and, for β-eudesmol, CYP3A4-mediated metabolism.

## 4. Conclusions

Overall, the results indicate that *T. polium* is a rich source of polyphenols and flavonoids with marked antioxidant capacity and that its essential oil contains a volatile fraction rich in limonene, α-muurolol and β-eudesmol with significant antifungal activity. Although the antibacterial MICs of the crude extracts and essential oil were generally high, checkerboard testing revealed selected synergistic and additive interactions between the essential oil and clinically used antibiotics against MDR isolates, confirming the concept of essential oil-derived adjuvants. Given the high concentrations required for direct inhibition, future developments should focus on standardized delivery systems (nanoemulsions and lipid carriers) designed for topical or localized administration, as well as pharmacokinetic and safety evaluation. In addition, the presence of terpenoids known or suspected to cause skin sensitization (particularly oxidized derivatives of limonene) highlights the need for targeted dermatological safety testing and monitoring of peroxide in any prospective skin formulation.

## Figures and Tables

**Table 1:** Origin, plant part used, collection site, and harvesting period of *T. polium*.

Latin Name	Part Used	Latitude (x)	Longitude (y)	Altitude (m)	Harvest Year	Voucher Number
*T. polium*	Flowering tops	34°30′07.3	4°09′24.4	558	May and June 2024	RAB115045

**Table 2:** List of bacterial strains tested.

Strain		Abbreviation	Reference
Gram-positive cocci	*Staphylococcus aureus* BLACT	*S. aureus* BLACT	4IH2510
	*Staphylococcus aureus*	*S. aureus*	2DT2220
	*Staphylococcus epidermidis*	*S. epidermidis*	5994
Gram-negative bacilli	*Acinetobacter baumannii*	*A. baumannii*	2356
	*Escherichia coli* ESBL	*E. coli* ESBL	2DT5765
	*Enterobacter cloacae*	*E. cloacae*	6412
	*Klebsiella pneumoniae* ssp. *pneumoniae*	*K. pneumoniae*	5694
	*Pseudomonas aeruginosa*	*P. aeruginosa*	5824
	*Enterobacter cloacae*	*E. cloacae*	02EV317
	*Escherichia coli*	*E. coli*	3DT1938
	*Klebsiella pneumoniae* ssp. *pneumoniae*	*K. pneumoniae*	3DT1823

**Table 3:** Antibiotics evaluated.

Antibiotic (ATB)	Abbreviation	Chemical Family
Gentamicin	GEN	Aminoglycosides
Vancomycin	VAN	Glycopeptides
Ceftazidime	CAZ	β-Lactams (3rd-generation cephalosporin)
Ceftriaxone	CRO	β-Lactams (3rd-generation cephalosporin)
Ciprofloxacine	CIP	Fluoroquinolone
Imipenem	IMP	Carbapenem
Amikacin	AN	Aminoglycosides
Ertapenem	ETP	Carbapenem

**Table 4:** Interpretation of the FICI.

Effect	FICI
Synergistic	≤0.5
Additive	0.5 < FICI ≤ 1
Indifferent	1 < FICI < 4
Antagonistic	≥4

**Table 5:** Protein targets and PDB codes.

Strain	Targets	PDB ID	Grid Center	Grid Size
*P. aeruginosa*	MexB (RND efflux pump)	2V50	center_x = 69.319 center_y = 30.691 center_z = −12.602	size_x = 34 size_y = 32 size_z = 40
	AmpC β-lactamase	4WZ4	center_x = −5.711 center_y = −13.034 center_z = 18.412	size_x = 30 size_y = 22 size_z = 16
*K. pneumoniae*	CTX-M β-lactamase (ESBL)	4S2I	center_x = 10.259 center_y = 13.254 center_z = 12.920	size_x = 24 size_y = 22 size_z = 16
	OmpK36 porin	6RD3	center_x = 8.031 center_y = −14.337 center_z = −8.017	size_x = 22 size_y = 26 size_z = 30
*E. coli*/ *E. cloacae*	AcrB (RND efflux pump)	2DHH	center_x = 149.827 center_y = 101.571 center_z = 39.139	size_x = 36 size_y = 32 size_z = 28
	TEM β-lactamase	1BTL	center_x = 5.264 center_y = 2.539 center_z = 32.763	size_x = 24 size_y = 20 size_z = 26
	Penicillin Binding Protein (PBP)	3OCL	center_x = 5.851 center_y = 4.924 center_z = −13.999	size_x = 22 size_y = 28 size_z = 22
*A. baumannii*	OXA-23 carbapenemase	4JF4	center_x = 11.525 center_y = −5.379 center_z = 4.630	size_x = 20 size_y = 26 size_z = 26
*S. aureus*	BlaZ (plasmid β-lactamase)	1BLZ	center_x = 10.886 center_y = 38.899 center_z = 4.89	size_x = 40 size_y = 20 size_z = 24

**Table 6:** Quality-control parameters of *T. polium* plant material.

Plant	Moisture Content (%)	pH	Ash (%)	Titratable Acidity (%)
*T. polium*	10.49 ± 0.15	5.46 ± 0.02	8.92 ± 0.09	10.25 ± 0.01

**Table 7:** Elemental concentrations in *T. polium* flowering tops (ICP–AES).

Element	As	Cr	Sb	Pb	Cd	Fe	Cu	Ti
Content (mg/kg DW)	0.0404	0.0054	0.0369	<0.0001	<0.0001	4.8004	0.1186	0.0322
Maximum limits (FAO/WHO mg/kg)	1	2	1	3	0.3	20	-	-

**Table 8:** Phytochemical screening of *T. polium* flowering tops.

Category	Chemical Groups			*T. polium*
Secondary metabolites	Tannins		Catechin tannins	+
			Gallic tannins	+
	Flavonoids			+
	Cyanidin reaction			Flavone
	Leucoanthocyanins			−
	Anthocyanins			+
	Saponins			+
	Alkaloids			−
	Reducing compounds			+
	Monosaccharides et holosides			+
	Mucilages			+
	Sterols and triterpenes			+
Primary metabolites	Polysaccharides			+
	Reducing sugars (glucose and fructose)			+
	Proteins	Biuret test		−
		xanthoproteic reaction		+
	Lipids (Liebermann–Burchard reaction)			+

(+): present; (−): absent.

**Table 9:** Organoleptic and physicochemical properties of *T. polium* EO.

Plant	Properties			
	Yield %	Density (g/mL, 20 °C)	Color	Odor
*T. polium*	0.72 ± 0.02	0.931 ± 0.04	Dark yellow	Strong aroma

**Table 10:** Chemical composition of *T. polium* EO (GC–MS).

N°	KI	Compound	Area (%)
1	930	α-Thujene	1.53
2	939	α-Pinene	7.89
3	975	Sabinene	1.73
4	979	β-Pinene	10.1
5	990	Myrcene	1.98
6	1017	α-Terpinene	0.38
7	1029	Limonene	24.13
8	1059	γ-Terpinene	0.86
9	1096	Linalool	0.49
10	1139	trans-Pinocarveol	1.15
11	1164	Pinocarvone	0.7
12	1177	Terpinen-4-ol	2.72
13	1195	Myrtenal	0.82
14	1229	cis-Carveol	0.72
15	1237	Pulegone	0.49
16	1243	Carvone	1.39
17	1343	Piperitenone	0.63
18	1376	α-Copaene	0.98
19	1419	(E)-Caryophyllene	2.84
20	1454	α-Humulene	0.82
21	1471	Dehydro-sesquicineole	2.38
22	1481	Germacrene D	2.09
23	1490	β-Selinene	1.74
24	1500	Bicyclogermacrene	0.52
25	1513	γ-Cadinene	1.51
26	1523	δ-Cadinene	3.44
27	1544	cis-Sesquisabinene hydrate	0.65
28	1549	Elemol	1.67
29	1563	(E)-Nerolidol	1.35
30	1583	Caryophyllene oxide	1.25
31	1642	3-iso-Thujopsanone	0.51
32	1646	α-Muurolol	8.1
33	1650	β-Eudesmol	10.48
34	1684	epi-α-Bisabolol	1.41
35	1736	Eremophilone	0.56
Monoterpene hydrocarbons			48.6%
Oxygenated monoterpenes			9.11%
Sesquiterpene hydrocarbons			13.94%
Oxygenated sesquiterpenes			28.36%
Total			100%

KI: Kovats retention index.

**Table 11:** Extraction yields and phenolic contents of *T. polium* extracts.

Extract	Extraction Yield (%)	Total Polyphenols (mg GAE/g Extract)	Total Flavonoids (mg QE/g Extract)	Catechin Tannins (mg CE/g Extract)
Decoction	20.98 ± 0.55	144.72 ± 6.70	9.82 ± 0.08	12.31 ± 0.23
Aqueous extract	23.91 ± 0.58	139.97 ± 0.96	11.94 ± 0.30	11.52 ± 0.22
Hydroethanolic extract	20.56 ± 0.99	196.21 ± 1.92	21.44 ± 0.38	10.10 ± 0.03

**Table 12:** Compounds identified by HPLC/UV–ESI–MS in *T. polium* extracts. (D: decoction; H.E.: hydroethanolic extract.)

N°	RT	Molecule	Class	Exact Weight	[M−H]^−^ (*m*/*z*)	Ion Fragments (*m*/*z*)	Area (%)	
							D.	H.E.
1	7.28	Caffeic acid	Phenolic acid	180	179.1	135-117	0.93	-
2	7.49	Cinnamic acid	Phenolic acid	148	147	147-103	0.80	-
3	7.74	Quinic acid	Organic acid	192	191.1	173-127	1.02	-
4	8.60	Vanillic acid	Phenolic acid	168	167	152-123-108	0.82	0.26
5	11.71	Myricetin	Flavonoid	318	317.1	287-179	0.72	0.15
6	12.75	Gallocatechin	Flavonoid	306	305.1	261-179-125	0.93	-
7	13.22	Khayanthone	Phenolic compound	570	569.1	507-465	1.97	2.46
8	13.43	4-Glucogallic acid	Tannin	332	330.9	313-271-169	-	0.85
9	14.24	Chlorogenic acid	Phenolic acid	354	353.1	191-179	4.32	0.21
10	14.73	Catechin 7-*O*-glucoside	Flavonoid	452	451.1	289-179-165	0.84	0.65
11	15.93	Naringenin 7-*O*-glucoside	Flavonoid	434	433.2	271-151	2.88	-
12	16.14	Cirsimaritin	Flavonoid	314	313.1	298-269	2.57	-
13	16.27	Teucardoside	Phenolic compound	490	489.2	327-309	-	0.59
14	16.53	Caffeoyl-feruloyltartaric acid	Phenolic acid	488	487.1	325-179	0.86	-
15	16.92	Tremuloidin	Phenolic compound	390	389.1	389-269	-	0.19
16	17.14	Loganic acid	Terpenoid	376	375.1	213-169-151	-	0.15
17	17.27	Catechin-3-O-rhamnoside	Flavonoid	436	435.2	435-289	12.11	-
18	17.93	Diferulic acid	Phenolic acid	386	385.1	193-178-149	1.76	0.56
19	18.84	Gallic acid 4-*O*-(6-galloylglucoside)	Tannin	484	483.1	331-271-125	1.42	-
20	19.75	Epicatechin	Flavonoid	290	289.1	245-179-151	38.63	56.91
21	20.52	Quercetin 3-*O*-glucoside	Flavonoid	464	463.1	301-179	-	3.38
22	20.60	Dihydrosamidine	Flavonoid	388	387.1	343-223-197	3.06	-
23	20.9	Acteoside	Phenolic compound	624	623.2	461-315-161	-	1
24	21.04	Wogonin 7-*O*-glucuronide	Flavonoid	460	459.1	283-240	2.24	2.6
25	21.24	Apigenin 7-*O*-glucoside	Flavonoid	432	431	269-151-117	2.09	-
26	21.43	Harpagide	Terpenoid	364	363	345-183-165	1.75	-
27	21.49	Luteolin 7-*O*-rutinoside	Flavonoid	594	593.1	593-285	-	2.43
28	21.85	3-Hydroxyflavone	Flavonoid	238	237	151-133	2.03	2.18
29	21.94	Epigallocatechin gallate	Flavonoid	458	457.1	331-169	-	1.58
30	22.29	Apigenin 7-*O*-glucuronide	Flavonoid	446	445.1	269-225-117	1.78	-
31	22.70	Salicortin	Phenolic compound	424	423.2	285-155-137	1.25	-
32	22.99	Rosmarinic acid	Phenolic acid	360	359.1	197-179-161-135	1.36	1.46
33	23.04	Rhamnetin	Flavonoid	316	315.2	300-165-151	-	1.03
34	23.37	Salvigenin	Flavonoid	328	327.2	312-283	1.08	1.67
35	23.43	3-Feruloyl-4-caffeoylquinic acid	Phenolic acid	530	529.1	367-191-135	-	0.83
36	23.75	7-Methoxy-2-methylisoflavone	Flavonoid	266	265.1	250-223	1.59	-
37	23.79	Retusin	Flavonoid	358	357.1	342-327	-	1.19
38	24.48	Resveratrol	Phenolic compound	228	227.1	185-143	0.87	-
39	25.15	Cirsilineol	Flavonoid	344	343.2	328-299-271	2.73	-
40	25.32	Luteolin	Flavonoid	286	285	175-133	4.08	4.61
41	25.57	Apigenin	Flavonoid	270	269	225-151-117	-	4.37
42	26.13	Emmotin H	Terpenoid	244	243.1	228-200-184	-	0.95
43	26.29	Cirsiliol	Flavonoid	330	329.1	314-285	1.53	3.6
44	26.88	Hydroxycaffeic acid	Phenolic acid	196	194.9	179-135	-	0.85
45	27.01	Luteolin 7-*O*-glucuronide	Flavonoid	462	461.3	285-175	-	1.55
46	27.38	N-Caffeoylputrescine	Hydroxycinnamic amide	250	249.1	179-135	-	0.53
47	27.70	Acacetin	Flavonoid	284	283.1	268-151	-	1.21

**Table 13:** Antioxidant activities of *T. polium* extracts and EO (different assays).

Extract	DPPH IC_50_ (µg/mL)	FRAP EC_50_ (µg/mL)	TAC (mg EAA/g)
Decoction	27.07 ± 1.38	51.72 ± 1.08	316.60 ± 2.15
Aqueous extract	23.12 ± 0.04	40.18 ± 1.95	294.79 ± 11.03
Hydroethanolic extract	15.41 ± 1.04	30.65 ± 0.69	368.47 ± 6.14
EO (mg/mL)	24.06 ± 0.09	-	-
Ascorbic acid	2.71 ± 0.03	11.90 ± 0.36	-

**Table 14:** IC_50_, EC_50_, and PAR values of *T. polium* extracts.

Authors	Locality	Extract	IC50 (µg/mL)	EC50	PAR
Our results	Taza, Morocco	Soxhlet, [EtOH-H_2_O]	15.41 ± 1.04	0.64 ± 0.04	155.74 ± 10.51
Our results	Taza, Morocco	Soxhlet, H_2_O	23.12 ± 0.04	0.96 ± 0.00	103.81 ± 0.18
Our results	Taza, Morocco	Decoction, H_2_O	27.07 ± 1.38	1.13 ± 0.06	88.66 ± 4.52
Stankovic et al., 2012 [85]	Suva Planina, Serbia	Maceration, H_2_O	56.4 ± 1.53	1.41 ± 0.04	70.92 ± 1.92
Stankovic et al., 2012 [85]	Suva Planina, Serbia	Maceration, MeOH	59.37 ± 0.76	1.48 ± 0.02	67.37 ± 0.86
Bendjabeur et al., 2018 [84]	Bouira, Algeria	Soxhlet, absolute EtOH	47.45 ± 0.29	1.98 ± 0.01	50.58 ± 0.31
Timizar et al., 2024 [54]	Bouira, Algeria	Soxhlet, EtOH 80%	65	2.03	49.23

**Table 15:** MIC (µg/mL) of terbinafine against the tested fungal strains.

Microorganism	Terbinafine
*Candida albicans*	12.500
*Candida dubliniensis*	3.125
*Candida tropicalis*	12.500
*Candida parapsilosis*	6.250
*Aspergillus niger*	3.125

**Table 16:** MIC (µg/mL) of antibiotics determined by BD Phoenix and VITEK2 for selected strains.

Microorganism	Reference	MIC (µg/mL) Identification and Antibiogram Instrument BD Phoenix™ VITEK2												
		GEN	AMC	VAN	SXT	IMP	AMP	CIP	CRO	ERY	TET	OXA	CAZ	LEV
*S. aureus* BLACT	4IH2510	<0.5		2	<10									
*S. aureus*	2DT2220	>8		>8	>4/76	>32				>8	>1	>4		
*S. epidermidis*	5994	2		>8	>4/76									
*E. cloacae*	02EV317	>4	>8/2		>4/76									
*Escherichia coli*	3DT1938	2	8/2		≤1/19									
*K. pneumoniae*	3DT1823	≤1	≤2/2		≤1/19									
*A. baumannii*	2356	>8	>32/2		>8/152	>8	>16	>1	>4				>16	>4
*E. coli* ESBL	2DT5765	>16	>32/2		>320	1	>32	>4	>64					
*E. cloacae*	6412	>8	>32/2		>8/152	4	>16	>1	>4				>16	>4
*K. pneumoniae*	5694	8	>32		40	8	>32	>4	>64					
*P. aeruginosa*	5824	>4	>8/2		>4/76	>8	>32	>4	>64					

GEN: gentamycin; AMC: amoxicillin–clavulanate; VAN: vancomycin; SXT: trimethoprim–sulfamethoxazole; IMP: imipenem; AMP: ampicillin; CIP: ciprofloxacin; CRO: ceftriaxone; ERY: erythromycin; TET: tetracycline; OXA: oxacillin; CAZ: ceftazidime; LEV; levofloxacin.

**Table 17:** MIC, MBC, and MFC (mg/mL) of *T. polium* EO and extracts.

Extract/Strain		Decoction		Hydroethanolic		Aqueous		EO	
		MIC	MBC/MFC	MIC	MBC/MFC	MIC	MBC/MFC	MIC	MBC/MFC
Bacteria	*Enterobacter cloacae* 02EV317	50	50	25	50	>50	>50	25	25
	*Klebsiella pneumoniae* 3DT1823	50	50	50	50	>50	>50	25	25
	*Escherichia coli sauvage* 3DT1938	50	50	50	50	>50	>50	25	25
	*Staphylococcus aureus* BLACT 41H2510	25	50	50	50	>50	>50	50	50
	*Staphylococcus epidermidis* 5994	50	50	50	50	>50	>50	25	25
Fungi	*Candida albicans*	12.5	12.5	12.5	12.5	12.5	25	4.69	9.38
	*Candida dubliniensis*	12.5	25	6.25	12.5	6.25	12.5	9.38	9.38
	*Candida tropicalis*	6.25	6.25	6.25	6.25	6.25	6.25	9.38	9.38
	*Candida parapsilosis*	50	50	12.5	12.5	25	50	9.38	9.38
	*Aspergillus niger*	50	50	50	50	50	50	1.17	2.34

**Table 18:** FICI values for combinations of *T. polium* EO with antibiotics (checkerboard assay).

Strain	Compounds		MIC (mg/mL)				FIC		FICI	Output
			Alone		In Combination					
	EO	ATBs	EO	ATBs	EO	ATBs	EO	ATBs		
*K. pneumoniae* 5694	*T. polium*	GEN	50	0.512	1.56	0.256	0.0312	0.5	0.5312	Additive
		IMP	50	<0.016	<0.39	<0.016	-	-	-	-
		CIP	50	1.024	50	2.048	1	2	3	Indifferent
		CRO	50	>2.048	50	>2.048	1		1	Additive
		AN	50	0.256	3.125	0.064	0.0625	0.25	0.3125	Synergistic
		ERT	50	<0.016	<0.39	<0.016	-	-	-	-
		CAZ	50	>2.048	50	>2.048	1	-	-	-
*P. aerogenosa* 5824		GEN	50	<0.016	<0.39	<0.016	-	-	-	-
		IMP	50	0.032	0.39	0.032	0.0078	1	1.0078	Indifferent
		CIP	50	<0.016	<0.39	<0.016	-	-	-	-
		CRO	50	1.024	50	1.024	1	1	2	Indifferent
		AN	50	<0.016	<0.39	<0.016	-	-	-	-
		ERT	50	0.128	25	0.128	0.5	1	1.5	Indifferent
		CAZ	50	2.048	50	>2.048	1	-	-	-
*A. baumannii* 2356		GEN	25	0.512	50	0.256	2	0.5	2.5	Indifferent
		IMP	25	0.256	6.25	0.128	0.25	0.5	0.75	Additive
		CIP	25	1.024	6.25	0.512	0.25	0.5	0.75	Additive
		CRO	25	>2.048	25	>2.048	1	-	-	-
		CAZ	25	1.024	3.125	0.512	0.125	0.5	0.625	Additive
*E. cloacae* 6412		GEN	50	>2.048	>50	>2.048	-	-	-	-
		IMP	50	0.256	25	0.128	0.5	0.5	1	Additive
		CIP	50	1.024	1.56	0.512	0.0312	0.5	0.5312	Additive
		CRO	50	>2.048	>50	>2.048	-	-	-	-
		AN	50	0.128	3.125	0.064	0.0625	0.5	0.5625	Additive
		ERT	50	0.128	12.5	0.256	0.25	2	2.25	Indifferent
		CAZ	50	>2.048	>50	>2.048	-	-	-	-
*S. aureus* BLACT 4IH2510		GEN	50	0.512	12.5	0.512	0.25	1	1.25	Indifferent
		IMP	50	<0.016	0.39	0.016	0.0078	-	-	-
		CIP	50	0.256	25	0.512	0.5	2	2.5	Indifferent
		CRO	50	2.048	25	0.256	0.5	0.125	0.625	Additive
		AN	50	2.048	>50	>2.048	-	-	-	-
		ERT	50	0.016	0.39	<0.016	0.0078	-	-	-
		CAZ	50	2.048	1.56	0.016	0.0312	0.0078	0.039	Synergistic
		VAN	50	2.048	>50	>2.048	-	-	-	-
*S. aureus* 2DT2220		GEN	50	2.048	>50	>2.048	-	-	-	-
		IMP	50	0.512	3.125	1.024	0.0625	2	2.062	Indifferent
		CIP	50	1.024	25	>2.048	0.5	-	-	-
		CRO	50	2.048	>50	>2.048	-	-	-	-
		AN	50	0.512	25	0.128	0.5	0.25	0.75	Additive
		ERT	50	0.128	25	0.256	0.5	2	2.5	Indifferent
		CAZ	50	>2.048	>50	>2.048	-	-	-	-
		VAN	50	>2.048	>50	>2.048	-	-	-	-
*E. coli* ESBL 2DT5765		GEN	50	2.048	>50	>2.048	-	-	-	-
		IMP	50	<0.016	0.39	<0.016	0.0078	-	-	-
		CIP	50	0.064	12.5	0.128	0.25	2	2.25	Indifferent
		CRO	50	0.128	0.78	0.064	0.0156	0.5	0.5156	Additive
		AN	50	>2.048	>50	>2.048	-	-	-	-
		ERT	50	<0.016	0.78	0.016	0.0156	-	-	-
		CAZ	50	1.024	0.78	0.128	0.0156	0.125	0.1406	Synergistic

**Table 19:** Binding affinity of antibacterial target proteins for the selected ligands (kcal/mol).

Compounds/Targets	2V50	4WZ4	4S2I	6RD3	3OCL	4JF4	1BLZ	1BTL	2DHH
α-Pinene	−6.8	−4.7	−4.8	−5.8	−5.2	−5.1	−5.7	−4.4	−5.9
β-Pinene	−6.7	−4.7	−4.9	−5.9	−5.1	−5.1	−5.8	−4.5	−5.4
Limonene	−7.1	−5.1	−4.8	−6	−5	−5.3	−5.9	−4.5	−7.3
α-Muurolol	−8.6	−6	−6	−7.5	−6.4	−6.3	−7.4	−5.5	−7.2
β-Eudesmol	−8.5	−6.6	−6.2	−7.5	−6.8	−6.7	−7.7	−6	−8.5

**Table 20:** Interactions between target proteins and ligands showing the highest binding affinity.

Target/Compound	α-Muurolol		β-Eudesmol	
	2D	3D	2D	3D
2V50	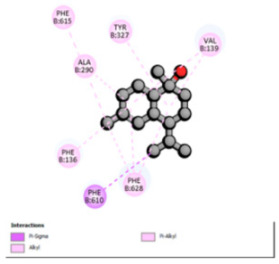	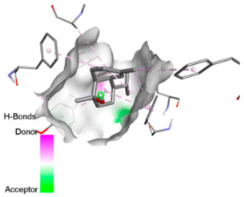	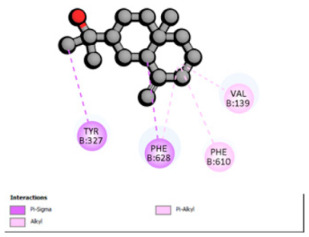	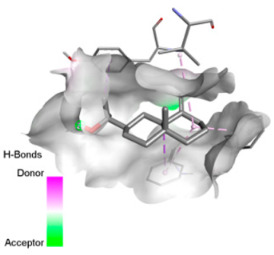
4WZ4	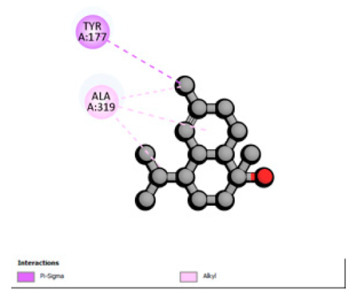	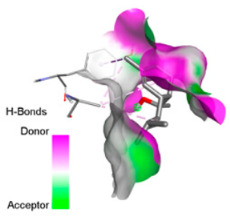	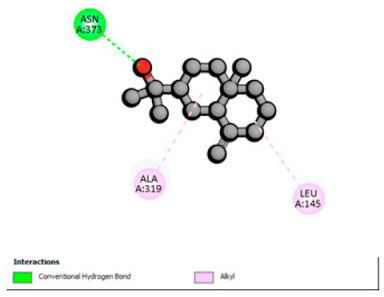	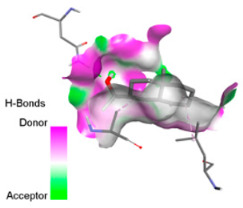
6RD3	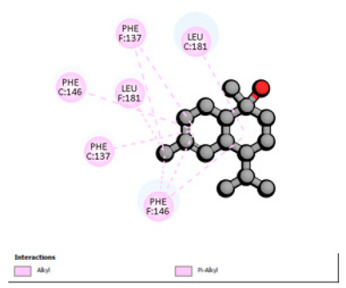	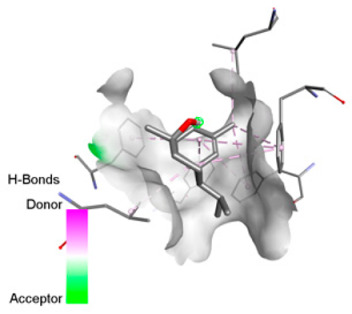	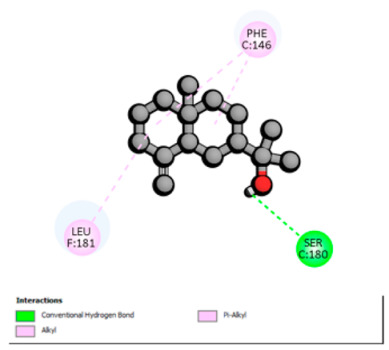	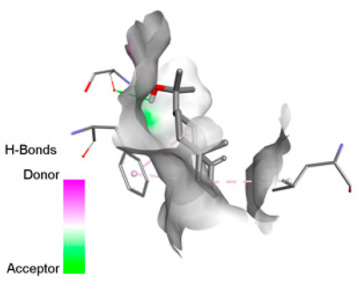
3OCL	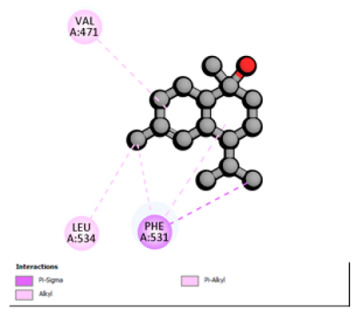	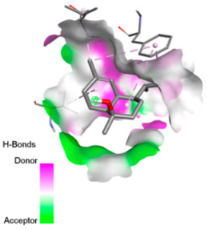	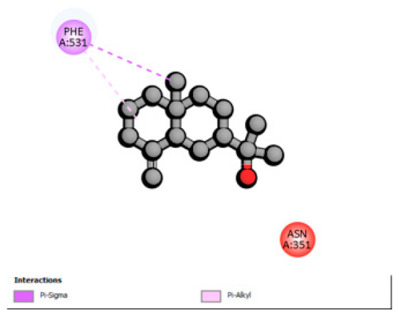	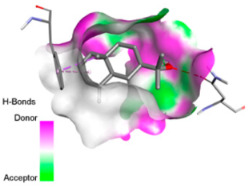
2DHH	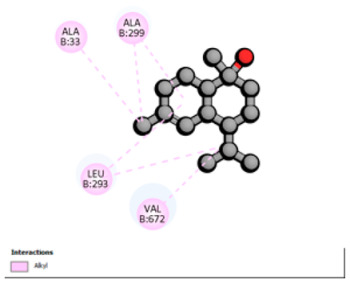	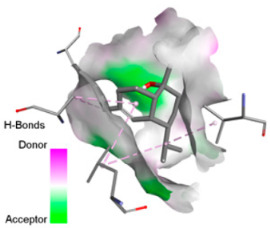	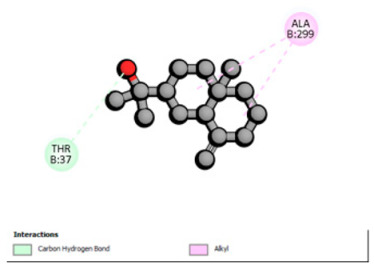	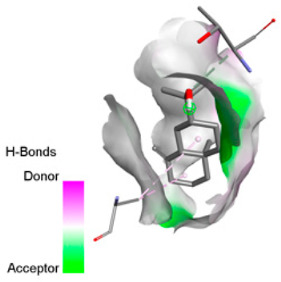
4JF4	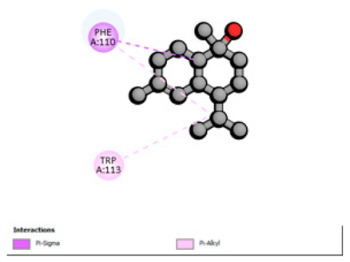	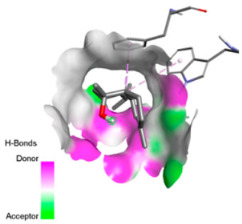	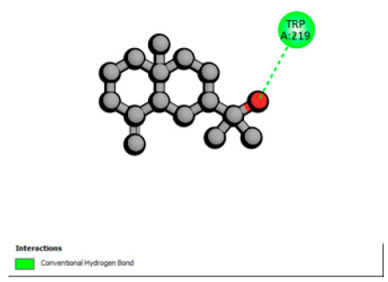	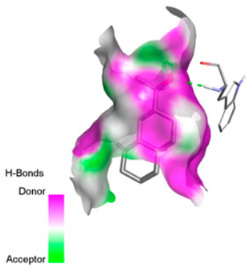
1BLZ	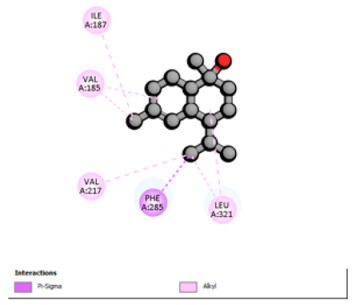	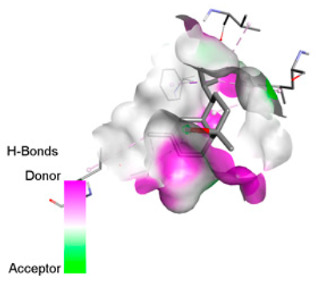	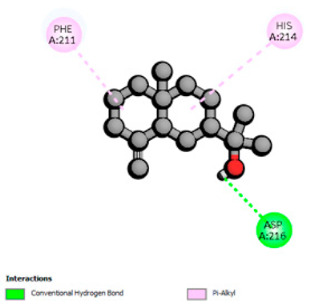	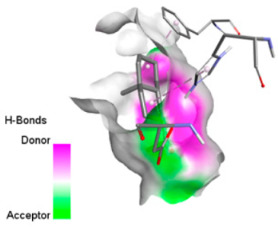

**Table 21:** In silico ADME analysis of *T. polium* EO constituents.

ADMET Properties	Compounds				
	α-Pinene	β-Pinene	Limonene	α-Muurolol	β-Eudesmol
General properties					
Molecular weight (g/mol)	136.23	136.23	136.23	222.37	222.37
Fraction Csp3	0.80	0.80	0.60	0.87	0.87
Rotatable bonds	0	0	1	1	1
H-bond acceptors	0	0	0	1	1
H-bond donors	0	0	0	1	1
Molecular refractivity	45.22	45.22	47.12	70.72	70.46
Topological polar surface area (Å2)	0.00	0.00	0.00	20.23 Å^2^	20.23
Lipophilicity Log Po/w	2.91	3.42	3.37	3.42	3.60
Water solubility Log S (Ali)	−2.23	−2.48	−2.26	−2.73	−3.21
Drug-likeness (Lipinski’s Rule)	Yes; 1	Yes; 1	Yes; 0	Yes; 0	Yes; 0
Bioavailability Score	0.55	0.55	0.55	0.55	0.55
Leadlikeness	No; 2	No; 2	No; 2	No; 1	No; 2
Absorption					
Water solubility (log mol/L)	Soluble	Soluble	Soluble	Soluble	Soluble
	−3.733	−4.191	−3.568	−4.073	−4.9
Caco2 permeability (log Papp in 10–6 cm/s)	1.38	1.385	1.401	1.479	1.508
GI absorption	Low	Low	Low	High	High
Skin permeability—log Kp (cm/s)	−1.827	−1.653	−1.721	−1.923	−1.967
P-glycoprotein substrate	No	No	Yes	No	No
P-glycoprotein inhibitor	No	No	No	No	No
Distribution					
VDss in humans (log L/kg)	0.667	0.685	0.396	0.42	0.459
Fraction unbound in humans (Fu)	0.425	0.35	0.48	0.28	0.164
BBB permeability (log BB)	0.791	0.818	0.732	0.596	0.634
CNS permeability (log PS)	−2.201	−1.857	−2.37	−2.151	−1.858
Metabolism					
CYP2D6 substrate	No	No	No	No	No
CYP3A4 substrate	No	No	No	No	Yes
CYP1A2 inhibitor	No	No	No	No	No
CYP2C19 inhibitor	No	No	No	No	No
CYP2C9 inhibitor	No	No	No	No	No
CYP2D6 inhibitor	No	No	No	No	No
CYP3A4 inhibitor	No	No	No	No	No
Excretion					
Total clearance (log mL/min/kg)	0.043	0.03	0.213	1.085	1.032
Renal OCT2 substrate	No	No	No	No	No

**Table 22:** In silico predicted toxicity of *T. polium* EO constituents (ProTox-II).

Parameters	Compounds				
	α-Pinene	β-Pinene	Limonene	α-Muurolol	β-Eudesmol
Max. tolerated dose (human) (log mg/kg/day)	0.48	0.371	0.777	0.343	−0.22
hERG I inhibitor	No	No	No	No	No
hERG II inhibitor	No	No	No	No	No
Oral rat acute toxicity (LD50) (mol/kg)	1.77	1.673	1.88	1.918	1.727
Oral rat chronic toxicity (LOAEL) (log mg/kg bw/day)	2.262	2.28	2.336	1.475	1.304
AMES toxicity	No	No	No	No	No
Hepatotoxicity	No	No	No	No	No
Skin Sensitization	No	No	Yes	Yes	Yes
T. pyriformis toxicity (log μg/L)	0.45	0.628	0.579	1.49	1.805
Minnow toxicity (log mM)	1.159	1.012	1.203	0.743	0.412
Predicted LD50 (mg/kg)	3700	4700	4400	2830	2000
Predicted Toxicity Class	5	5	5	5	4

## Data Availability

Data are contained within this article.
